# Label-free microfluidic sorting of microparticles

**DOI:** 10.1063/1.5120501

**Published:** 2019-12-11

**Authors:** Jian Zhou, Prithviraj Mukherjee, Hua Gao, Qiyue Luan, Ian Papautsky

**Affiliations:** Department of Bioengineering, University of Illinois at Chicago, Chicago, Illinois 60607, USA

## Abstract

Massive growth of the microfluidics field has triggered numerous advances in focusing, separating, ordering, concentrating, and mixing of microparticles. Microfluidic systems capable of performing these functions are rapidly finding applications in industrial, environmental, and biomedical fields. Passive and label-free methods are one of the major categories of such systems that have received enormous attention owing to device operational simplicity and low costs. With new platforms continuously being proposed, our aim here is to provide an updated overview of the state of the art for passive label-free microparticle separation, with emphasis on performance and operational conditions. In addition to the now common separation approaches using Newtonian flows, such as deterministic lateral displacement, pinched flow fractionation, cross-flow filtration, hydrodynamic filtration, and inertial microfluidics, we also discuss separation approaches using non-Newtonian, viscoelastic flow. We then highlight the newly emerging approach based on shear-induced diffusion, which enables direct processing of complex samples such as untreated whole blood. Finally, we hope that an improved understanding of label-free passive sorting approaches can lead to sophisticated and useful platforms toward automation in industrial, environmental, and biomedical fields.

## I. INTRODUCTION

Particle sorting is a critical step in numerous industrial, research, and biomedical applications.^1–4^ For instance, in mining and petroleum industries, microparticle separation is strongly associated with the economic value of end products.^4,5^ Separation of microparticles from cosmetics is important for quality control and regulation enforcement.^6^ It is also an indispensable step in environmental assessment of microplastics and nanoparticles.^7–9^ With the emergence of microfluidics, increasing interest in biomedical applications^10–12^ (e.g., diagnostics, therapeutics, and cell biology) has fueled the development of separation of biological microparticles, including cells,^13,14^ bacteria,^15,16^ extracellular vesicles (EVs),^17,18^ and even macromolecules such as deoxyribonucleic acid (DNA).^19,20^ In particular, the enormous clinical implications of circulating tumor cells (CTCs)^21,22^ and circulating EVs (e.g., exosomes^23,24^) for liquid biopsy in cancer diagnostics and treatment have been driving the burgeoning development of microfluidic devices for microparticle separation in recent years. In turn, microparticles are robust surrogates for bioparticles that have been extensively used for prototyping novel microfluidic devices and improving their separation performance.

A wide range of devices has been introduced for microfluidic sorting of microparticles. Owing to their small size and laminar flow nature, these devices are inherently capable of manipulation of fluid and suspended particles with remarkable spatial and temporal precision.^25^ Precise manipulation of the particle position inside microscale flow enables highly efficient sorting of particles if differential markers exist. Both biophysical and biochemical properties of the particles are widely exploited as markers for generating differentiated spatial distribution of particles inside microfluidic devices by adding either external or internal differentiating fields. Magnetic,^26–28^ electrical,^29–31^ acoustic,^32–34^ and optical^35–42^ forces are commonly used for differentiating particles flowing in a microfluidic channel. Such microfluidic devices typically offer precise, on-demand control of particle spatial distribution and are generally viewed as active methods of particle separation. This is because control of these forces as well as sophisticated device architecture is required. Table I lists the most common techniques in this category.

**TABLE I.** Summary of microfluidic platforms for sorting microparticles.

Active	Passive
Acoustophoresis	Inertial microfluidics (iMF)
Electrophoresis	Pinched flow fractionation (PFF)
Dielectrophoresis	Hydrodynamic filtration (HDF)
Magnetophoresis	Cross-flow filtration (CFF)
Optical tweezers	Deterministic lateral displacement (DLD)
Centrifugation	Gravity-driven separation^a^
	Viscoelastic microfluidics
	Shear induced diffusion (SID)

^a^ Active method without control, acting like the passive method.

Conversely, spatial differentiation of particles can be achieved by taking advantage of hydrodynamic forces due to the physical structure of the microfluidic channel or the intense interaction between particles suspended in flow. Since no external force field is necessary, these microfluidic approaches are termed passive separation methods (Table I). Passive methods are attractive alternatives due to their simplicity and low cost, with most methods also being label-free. Using these techniques, particles are distinguished and sorted according to their physical properties (size, density, shape, and deformability), making labor-intensive and time-consuming labeling steps (e.g., immunolabeling with magnetic beads) unnecessary. Some of the most prominent techniques in this group include inertial microfluidic (iMF) separation,^43–45^ pinched flow fractionation (PFF),^46^ hydrodynamic filtration (HDF),^47^ crossflow filtration (CFF),^48^ and deterministic lateral displacement (DLD).^49^

The purpose of this review is to provide an updated discussion of the state-of-the-art microfluidic devices developed for passive label-free particle separation. Due to the fast-growing interest and the still unmet need of particle separation in industrial, environmental, and biomedical applications, new microfluidic devices are being developed at an unprecedented rate. There are multiple reviews covering or touching upon this topic already. For example, McGrath *et al.* reviewed the evolvement and application of DLDs. The current understanding and applications of inertial microfluidics for separation were previously reviewed in 2014^50,51^ and in 2016.^52^ Discussion of PFF, CFF, and HDF was partly included in Pamme's early review.^53^ Recently, Sajeesh and Sen discussed these label-free microfluidic devices in a review of both passive and active methods for particle separation.^54^ However, most of these label-free methods were reviewed in the context of bioparticles, such as rare cells, due to the outstanding interest of separation of cellular components from bodily fluids such as blood.^10–12,55,56^ Further, these reviews are focused either narrowly on specific separation technics or on a broad coverage of common separation methods. Performance and applications of microfluidic devices of the same method are generally provided in these reviews, but cross-comparison among different methods is less detailed and the newly emerged approaches such as particle separation using shear-induced diffusion (SID)^57,58^ or viscoelastic flow are either not included or discussed only briefly.

**FIG. 1.**
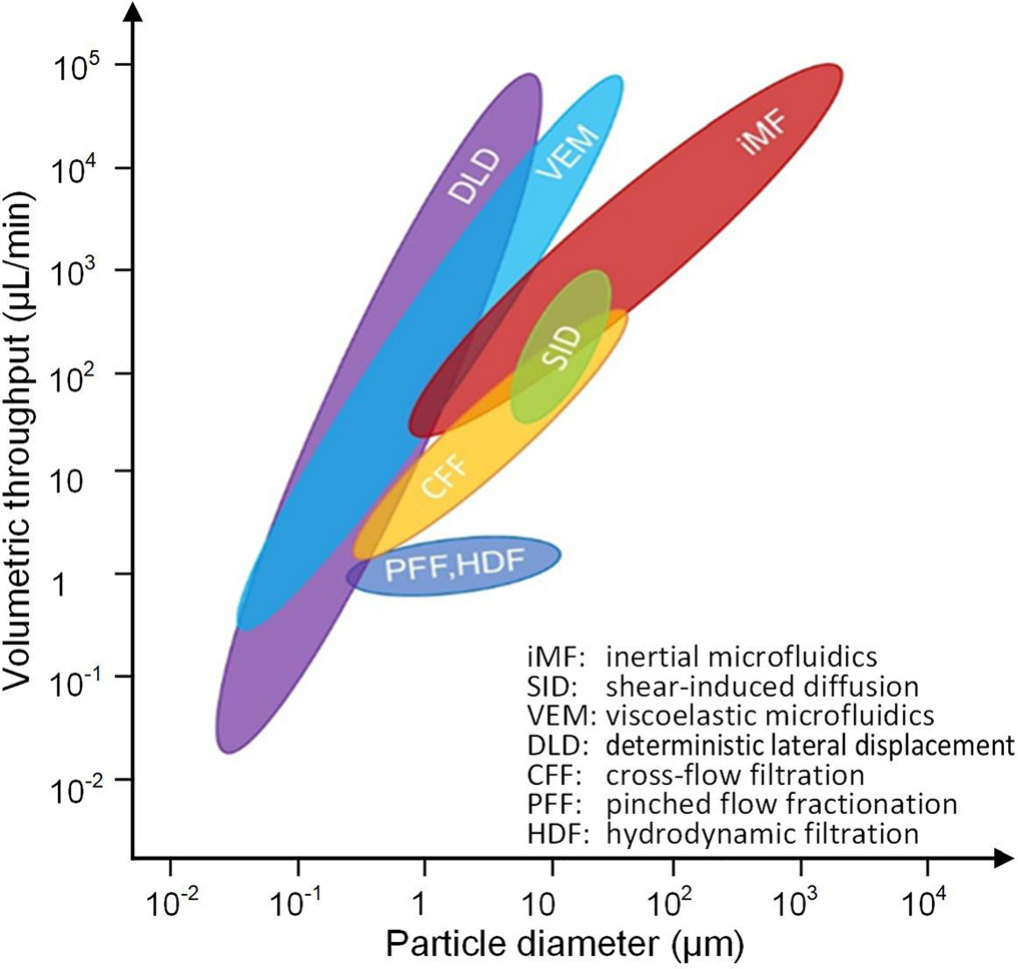
The passive label-free sorting methods in terms of volumetric throughput and microparticle diameter.

This review is focused on the label-free separation of particles in passive microfluidic devices, with emphasis on performance and operational conditions. As a group, these devices are capable of processing particles from the macro all the way down to the nanorange, with throughputs from nanoliters per minute to milliliters per minute or higher. Figure 1 graphically illustrates the performance range for each technique in terms of throughput and particle size. For each method, we first give a brief introduction to its working mechanism, followed by discussion of its variant designs and performance metrics. In addition to the now common separation approaches using Newtonian flows, such as DLD, PFF, CFF, HDF, and iMF, separation employing non-Newtonian viscoelastic flow will also be discussed. We also include the newly emerged SID method, which is capable of direct processing complex samples such as untreated whole blood.^57,58^ In the concluding section, comparison and discussion of the reviewed methods will be presented along with perspectives on future developments.

## II. SORTING BY INERTIAL MIGRATION

**FIG. 2.**
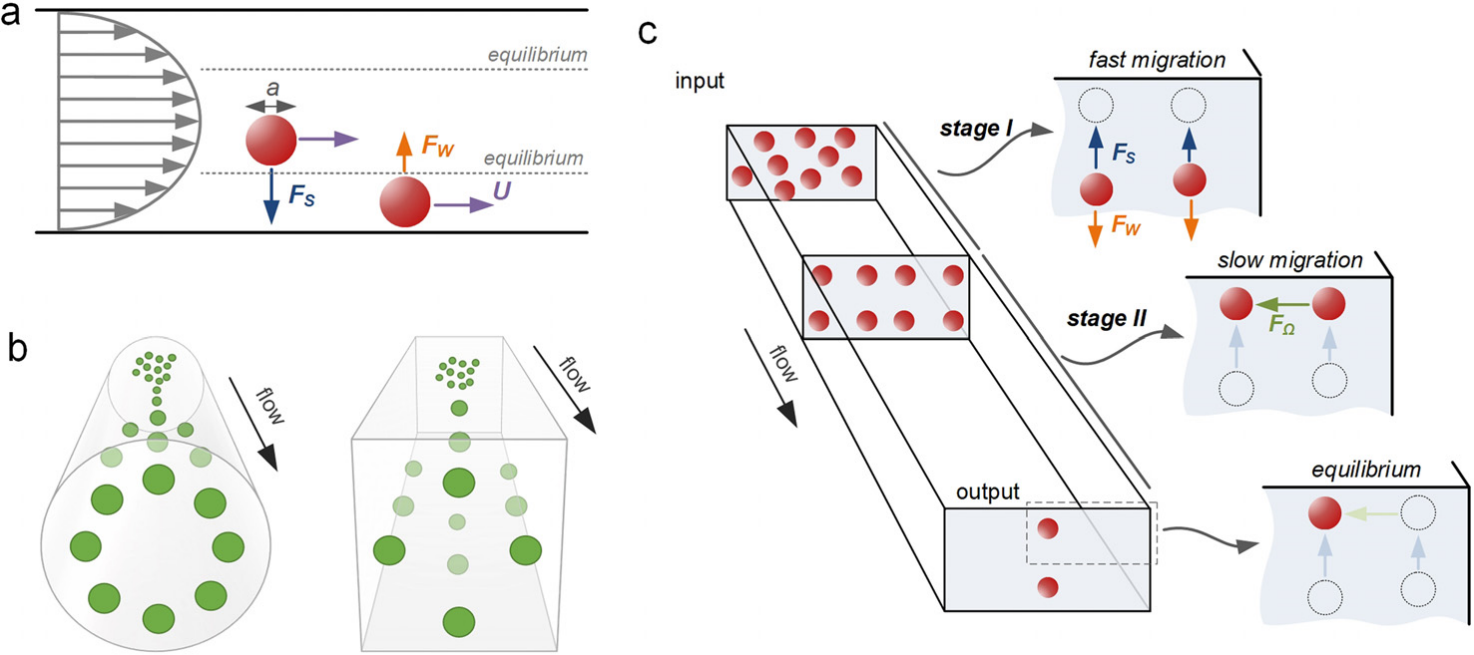
Inertial focusing in microchannels. (a) Two lift forces orthogonal to the flow direction act to equilibrate microparticles near the wall at *Re_p_* ≥ 1. The shear-induced lift force *F_s_* is directed down the velocity gradient and drives particles toward channel walls. The wall-induced lift force *F*_w_ directs particles away from the walls and drives particles toward the channel centerline. The balance of these two lift forces causes particles to equilibrate. (b) Inertial focusing creates an annulus in cylindrical capillaries and four symmetric positions in square channels. In both cases, the equilibrium positions are approximately at ∼0.4D_h_ from the sidewall. (c) In a rectangular channel, there are two preferred equilibrium positions. A two-stage model describes inertial migration of microparticles first from the channel bulk toward equilibrium positions near long walls under the influence of *F*_s_ and *F*_w_ and then parallel to channel walls into wall-centered equilibrium positions under the influence of the rotation-induced lift force (*F*_Ω_).^43^ Reproduced with permission from Zhou *et al.*, Lab on a Chip **13**, 1121–1132. Copyright 2013 Royal Society of Chemistry.

Label-free sorting of microparticles can be accomplished in microfluidic channels using inertia of fluid surrounding microparticles. In this approach, inertial effects drive microparticles across flow streamlines into equilibrium positions. It is well accepted now that inertial focusing of particles occurs when the particle Reynolds number *Re_p_* ≥ 1^50^ [*Re_p_* = *Re*(*a*/*D_h_*)2 = *ρU_f_a*2/*μD_h_*, where *Re* is the channel Reynolds number, *U_f_* is the average fluid flow velocity, *a* is the microparticle diameter, *ρ* is the fluid density, *μ* is the fluid viscosity, and *D_h_* is the hydraulic diameter of the channel]. As particles flow downstream, they experience shear-induced lift force *F_s_* induced by fluid shear as well as wall-induced lift force *F_w_* generated by the interaction of particles and channel walls. These forces scale strongly with the particle diameter, with the total net lift force *F_L_* acting on particles as *F_L_* ∝ *ρU_f_*^2^*a*^2^/*D_h_*^2^ near the channel center and as *F_L_* ∝ *ρU_f_*^2^*a*^6^/*D_h_*^4^ near the channel wall.^50^ Consequently, microparticles migrate across flow streamlines toward the equilibrium positions, approximately ∼0.2*D_h_* away from the channel sidewall, where the two forces balance each other, and the total net lift force *F_L_* becomes zero [Fig. 2(a)].

The number and location of these equilibrium positions are dependent on the microchannel geometry and its cross-sectional shape [Figs. 2(b) and 2(c)]. Inertial focusing of microparticles can be accomplished in straight, curved, or microvortex channels, as we discuss below. The microchannel cross section is generally rectangular, with either a low or high aspect ratio (*AR* = *h*/*w*), although trapezoidal and triangular cross-sectional channels have also been reported.

The ratio of the microparticle diameter to the size of the channel plays a key role in the focusing behavior. This ratio, sometimes termed blockage ratio or confinement ratio β = *a*/*D_h_*, is generally expected to be β > 0.07.^59^ Earlier work by Chun and Ladd^60^ showed preferential focusing for particles with β > 0.1 and was later confirmed by Di Carlo *et al.*^61,62^ and Bhagat *et al.*^59,63^ At lower confinement ratios, microparticles are too small to be significantly impacted by the inertial lift forces. Focusing behavior is also impacted by the volume fraction of the particles in the suspension. The volume fraction is generally restricted to <1%;^43,61^ otherwise, particle-particle interactions disrupt the focusing.^44,64^

In this sorting approach, size differences between microparticles cause migration to distinct equilibrium positions within the channel cross section and at different rates (with larger particles migrating faster). This leads to two general types of devices for sorting of microparticles, some that amplify small spatial differences between equilibrium positions of differently sized microparticles and others that take advantage of differences in the migration rate. As Fig. 1 illustrates, collectively, these devices are capable of sorting particles in the micrometer to millimeter range, with throughputs from tens of microliters per minute to tens of milliliters per minute.

### A. Straight channels

The simplicity of straight channels makes them an ideal geometry for investigating the underlying physics of inertial migration and for sorting particles. The earlier seminal work by Segré and Silberberg^65,66^ showed that inertial migration causes particles to form an annulus in a fully symmetrical circular capillary approximately ∼0.2*D* from the sidewall [Fig. 2(b)]. Later, numerical studies by Chun and Ladd^60^ and experimental studies by Kim and Yoo^67^ and our group^59^ demonstrated four equilibrium positions in a square channel [Fig. 2(b)], which are further reduced to two in a rectangular channel at moderate *Re*. Fluid inertia surrounding particles is responsible for their cross-stream migration and predictable equilibration. Inertial forces including shear-induced lift force (*F_s_*) and wall-induced lift force (*F_w_*) are generally considered to be dominant, dictating particle migration dynamics. However, the full understanding of such a phenomenon remains to be achieved.

Our group proposed a two-stage migration model for particle focusing dynamics in a straight channel [Fig. 2(c)].^43^ Using a pair of straight rectangular channels with reciprocal aspect-ratio (AR), we comprehensively investigated inertial focusing behavior to explain the occurrence of two equilibrium positions. We showed experimentally the role of rotational lift force (*F*_Ω_) in the inertial migration of particles. In stage I, particles migrate to the top and bottom walls under the influence of shear gradient lift or negative lift (*F_L_*^−^), whereas in stage II, particles migrate to the center of the top and bottom channels under the influence of rotation induced lift or positive lift (*F_L_^+^*). By combining the expressions of Stokes' drag (*F_D_* = 3*πμaU*), Shear rate (*G *=* *2*U_f_*/*D_h_*), and particle lateral migration velocity (*U_L_* = 4*ρC_L_ U_f_*^2^*a*/3*πμ D_h_*^2^), we were able to calculate and experimentally determine the lift coefficients (C_L_), which was previously only possible with numerical simulations.

**FIG. 3.**
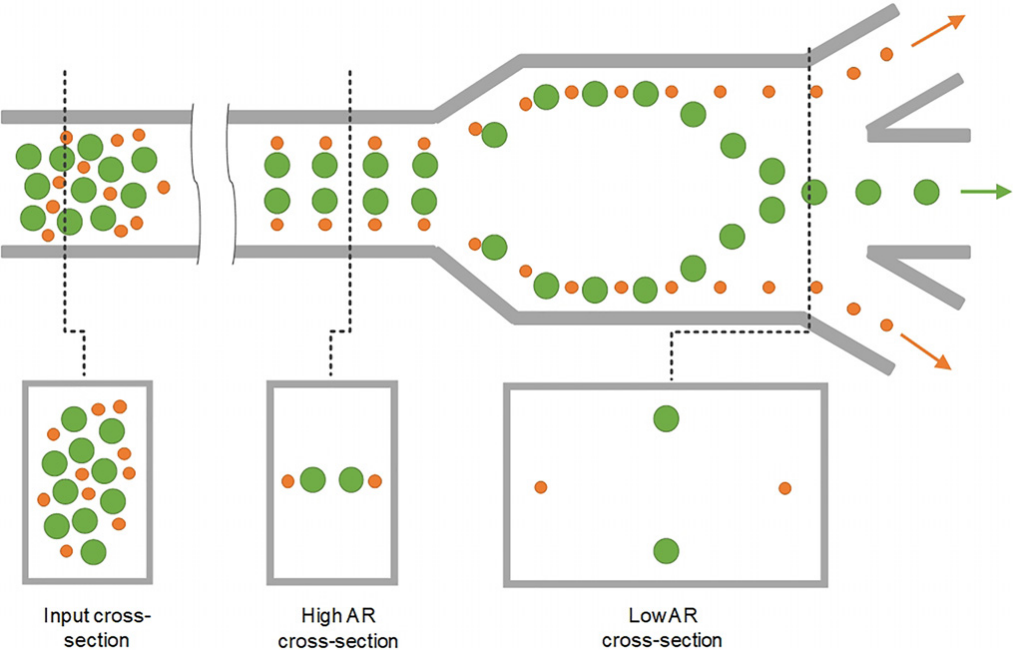
Representative design of straight inertial microfluidic devices for microparticle separation. Complete particle separation in the straight channel by switching the aspect ratio.^44^ Particle positions are also shown in the cross-sectional view.

**FIG. 4.**
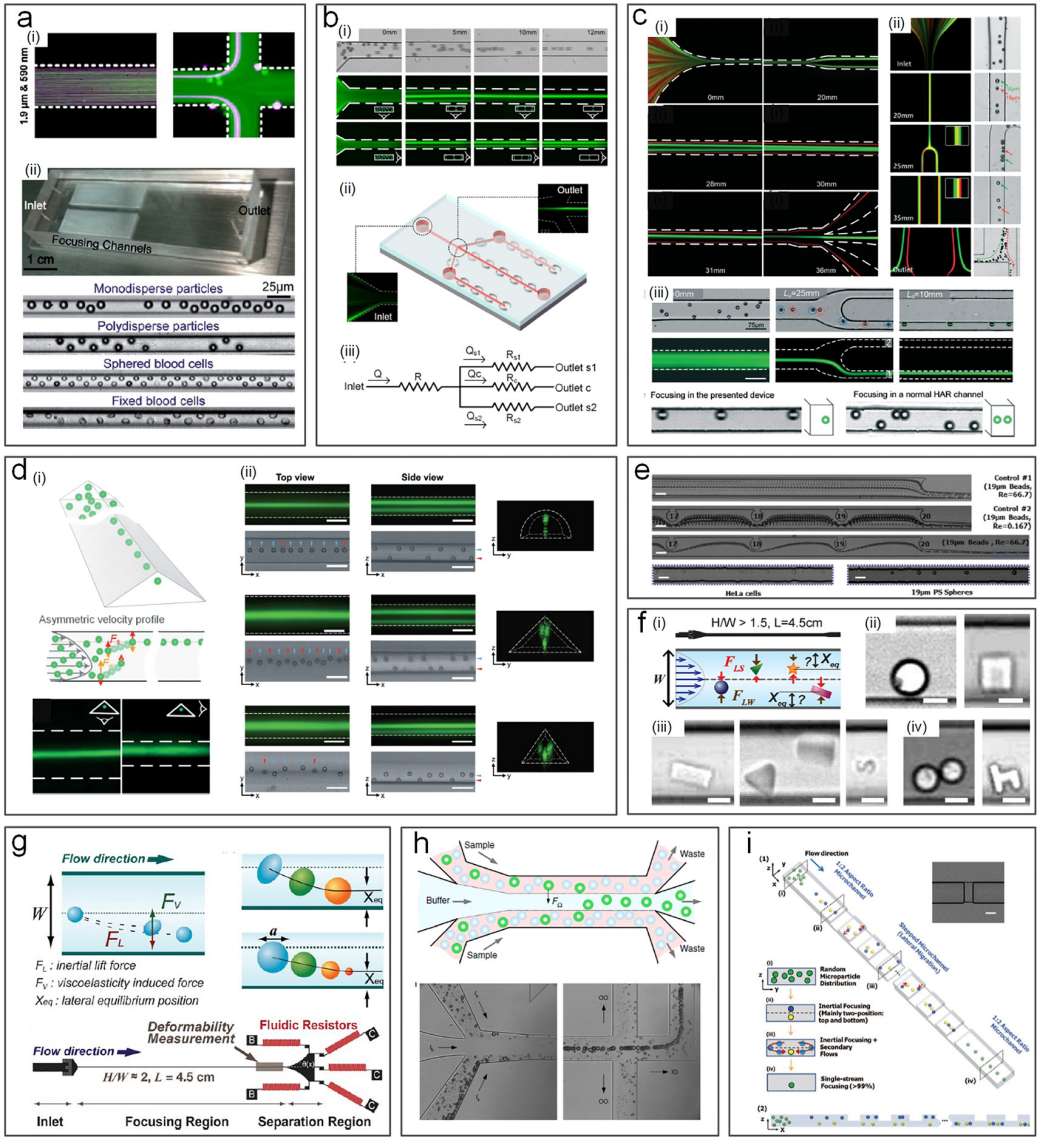
Inertial microfluidics for particle separation in straight channels. (a) High aspect-ratio (AR) straight channels employing Stage-I migration used for particle separation^68^ (i) and cell focusing^69^ (ii). Reproduced with permission from Bhagat *et al.*, Microfluid. Nanofluid. **7**, 217–226 (2009). Copyright 2009 Springer Nature.^68^ Reproduced with permission from Hur *et al.*, Lab on a Chip **10**, 274–280 (2010). Copyright 2010 Royal Society of Chemistry.^69^ (b) Low AR channel employing stage-I and stage-II migration for particle focusing^71^ (i) and for automatically tuning the cell concentration^45^ (ii). Reproduced with permission from Zhou *et al.*, Biomicrofluidics **8**, 044112 (2014). Copyright 2014 AIP Publishing.^71^ Reproduced with permission from Tu *et al.*, Biomed. Microdevices **19**(4), 83 (2017). Copyright 2017 Springer Nature.^45^ (c) Modulation of the channel aspect ratio based on the two-stage migration model for complete particle separation and single-stream focusing: (i) high AR → low AR,^44^ (ii) low AR → low AR,^73^ and (iii) low AR → high AR.^72^ Reproduced with permission from Zhou *et al.*, Lab on a Chip **13**(10), 1919–1929 (2013). Copyright 2013 Royal Society of Chemistry.^44^ Reproduced with permission from Wang *et al.*, Lab on a Chip **16**(10), 1821–1830 (2016). Copyright 2016 Royal Society of Chemistry.^73^ Reproduced with permission from Wang *et al.*, Lab on a Chip **15**(8), 1812–1821 (2015).^72^ Copyright 2015 Royal Society of Chemistry. (d) Single-^74^ and multiple-^75^ stream focusing of particles observed in triangular straight channels. Reproduced with permission from Mukherjee *et al.*, Lab on a Chip **19**(1), 147–157 (2019).^74^ Copyright 2019 Royal Society of Chemistry. Reproduced with permission from Kim *et al.*, Lab on a Chip **16**, 992–1001 (2016). Copyright Royal Society of Chemistry. Effects of channel microstructures^78^ (e), particle shape^80^ (f), and particle deformability^82^ (g) on inertial focusing have also been investigated. Reproduced with permission from Chung *et al.*, Lab on a Chip **13**(15), 2942–2949 (2013).^78^ Copyright 2013 Royal Society of Chemistry. Reproduced with permission from Hur *et al*., Lab on a Chip **11**(5), 912–920 (2011).^82^ Copyright 2013 Royal Society of Chemistry. Reproduced with permission from Hur *et al.*, Appl. Phys. Lett. **99**(4), 044101 (2011). Copyright 2011 AIP Publishing.^80^ (h) Particle and circulating tumor cell (CTC) separation achieved in an inertial coflow channel.^13^ Reproduced with permission from Zhou *et al.*, Microsyst. Nanoeng. **5**(1), 8 (2019). Copyright 2019 Authors licensed under a CC BY 4.0.^13^ (i) Secondary flow induced by the obstacles used for single stream particle focusing in a straight channel.^84^ Reproduced with permission from Chung *et al.*, Small **9**(5), 685–690 (2013). Copyright 2013 John Wiley and Sons.

This two-stage model of inertial migration is practically useful in design microchannels for microparticle focusing and separation. The model offers an expression for calculating the length of migration to full equilibrium: $L=\frac{3\pi \mu D_{h}^{2}}{4\rho U_{f}a^{3}}\left[\frac{h}{C_{L}^{-}}+\frac{w}{C_{L}^{+}}\right]$, where *w* is the longer and *h* is the shorter channel dimension. The channel length required for first stage or second stage focusing can be readily calculated and used in design of separation devices based on either high or low or hybrid aspect ratio channels (Fig. 3). A number of new designs have emerged in recent years, with either stage exploited for high-performance separation. Devices using stage I are typically high AR straight channels, permitting fast sample filtration^59,68–70^ [Fig. 4(a)]. On the other hand, filtration and concentration have been achieved using stage II migration in low AR straight channels^45,71^ [Fig. 4(b)]. Adding a buffer in the channel, a separation efficiency of ∼100% and a purity >87% were accomplished in our recent work.^13^ Changing the channel AR, our other work^44^ [Fig. 4(c-i)] readily achieved an efficiency >99% and a purity >90% without buffer flow. Following the two-stage model, our group has also successfully demonstrated “single-stream” focusing in straight channels using a low AR focusing channel followed by a bifurcation into additional low AR segments^72^[Fig. 4(c-iii)]. By replacing the second segments with low AR channels, 15 *μ*m and 18 *μ*m particles were separated with an efficiency^73^ >97% [Fig. 4(c-ii)].

Although channels with a rectangular cross section are commonly used for inertial focusing, other shapes of the channel cross section have also been reported. Recently, our group^74^ has demonstrated the focusing of particles in channels with a triangular cross section [Fig. 4(d-i)]. We successfully demonstrated single stream particle 3D focusing of 15 *μ*m in low-aspect ratio triangular channels. Kim *et al.*^75,76^ have also demonstrated single stream 3D focusing using a combination of rectangular, semicircular, and triangular channels [Fig. 4(d-ii)].

Other modifications to the channel internal structure and additional properties of suspended particles have also been explored for sorting purpose. Amini *et al.*^77^ added pillar microstructures within the straight microchannel, achieving the effect of stream sculpting, which assisted in the separation of 1 and 10 *μ*m diameter particles. Asymmetrical arrangement of the pillars near one side of the channel was later shown to be beneficial for fast particle migration^78^ [Fig. 4(e)]. The same group also investigated the effect of the particle shape as well as particle deformability if cells are concerned for preferential migration and separation^79–82^ [Figs. 4(f) and 4(g)]. Masaeli *et al.*^79^ reported separation of spheres from rod shaped particles with aspect ratios of 3:1 and 5:1. Introducing a buffer flow in the middle of a low AR channel was recently demonstrated in high-purity separation of particles and high-efficiency isolation of CTCs from blood [Fig. 4(h)].^13^ Additionally, the concentration of particles may also affect their migration and focusing in the way of particle-particle interaction dislodging the already focused particles.^83^ As a result, generally, the particle concentration is adjusted to less than 1% for inertial applications.

Inertial migration of microparticles results in hydrodynamic 3D confinement, which can be utilized for sheathless flow cytometry. Hur *et al.*^69^ demonstrated an inertial microfluidic device for sheathless flow cytometry and counting of erythrocytes and leukocytes with nearly 90% specificity. Chung *et al.*^84^ later designed another device, which combined inertial effects in a straight channel with the 3D-step induced helical secondary flows [Fig. 4(i)]. They were able to focus 9.9 *μ*m beads, achieving a focusing efficiency >99% at a throughput of 36 000 particles/s. Particle counting was also reported in other inertial devices including staged,^85^ spiral,^86^ bifurcation,^87^ and triangular^74^ channels.

Ultimately, one of the key advantages of the straight channels over the curvilinear and vortex channels discussed below is that straight channels can be paralleled to increase throughput tremendously. For example, a multiplexed array of 256 parallel channels was demonstrated to offer a throughput of 1.2 ml/min^69^ and filtration of 10 *μ*m particles from the mixture was achieved using a device with 16 channels.^88^

### B. Curvilinear channels

**FIG. 5.**
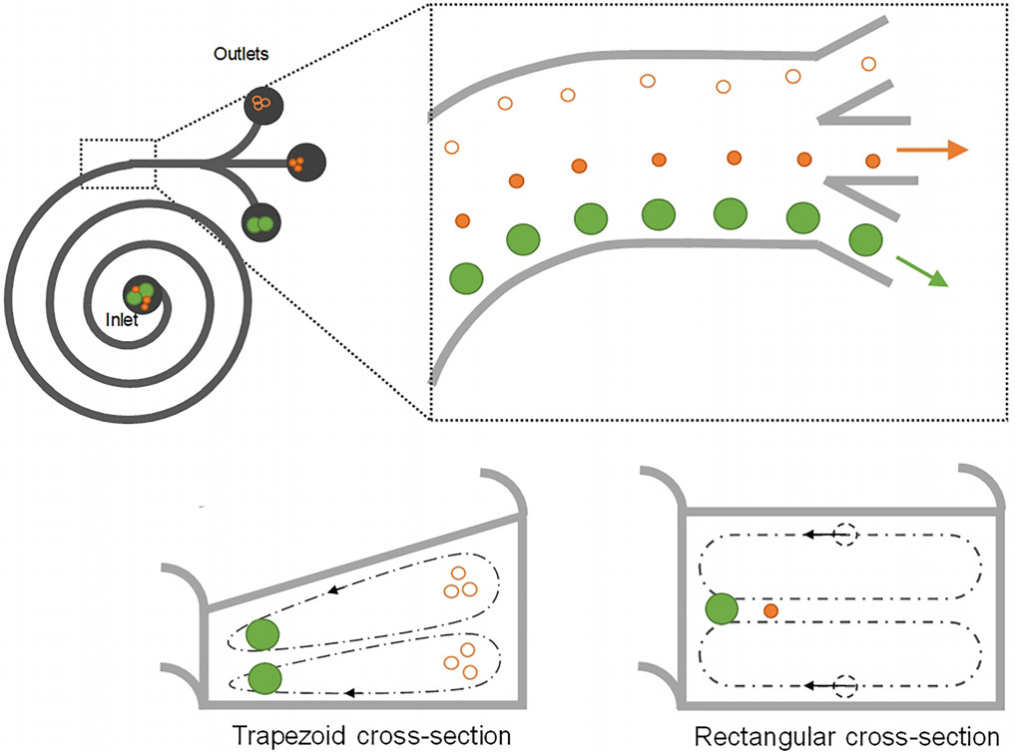
Schematic of spiral inertial microchannels. Spiral channel inducing counter-rotating secondary flows for particle separation.^63,89^ Distinct focusing positions have been proposed for channels with trapezoid^103,105^ and rectangular cross sections.^63,89^

In a curved microchannel, fluid undergoes centrifugal acceleration directed radially outward, leading to the formation of two counter-rotating vortices known as Dean vortices.^63,89^ The magnitude of Dean flow is given by a nondimensional parameter Dean number (*De*) as $De=Re\sqrt{\frac{D_{h}}{2R}}=\frac{\rho U_{f}D_{h}}{\mu }\sqrt{\frac{D_{h}}{2R}}$, where *R* is the radius of curvature. Particles flowing near the top or bottom of the channel cross section are subjected to Dean drag force *F_D_*, while the inertial lift forces are orthogonal, causing them to migrate with the Dean vortices. Near the outer wall, the net lift force *F_L_* is in the same direction as *F_D_*, and thus, particles follow the Dean vortices independent of their size. Near the inner wall, however, inertial and Dean forces act in opposite directions, leading to a possible force balance for particle focusing into a single position (Fig. 5).

**FIG. 6.**
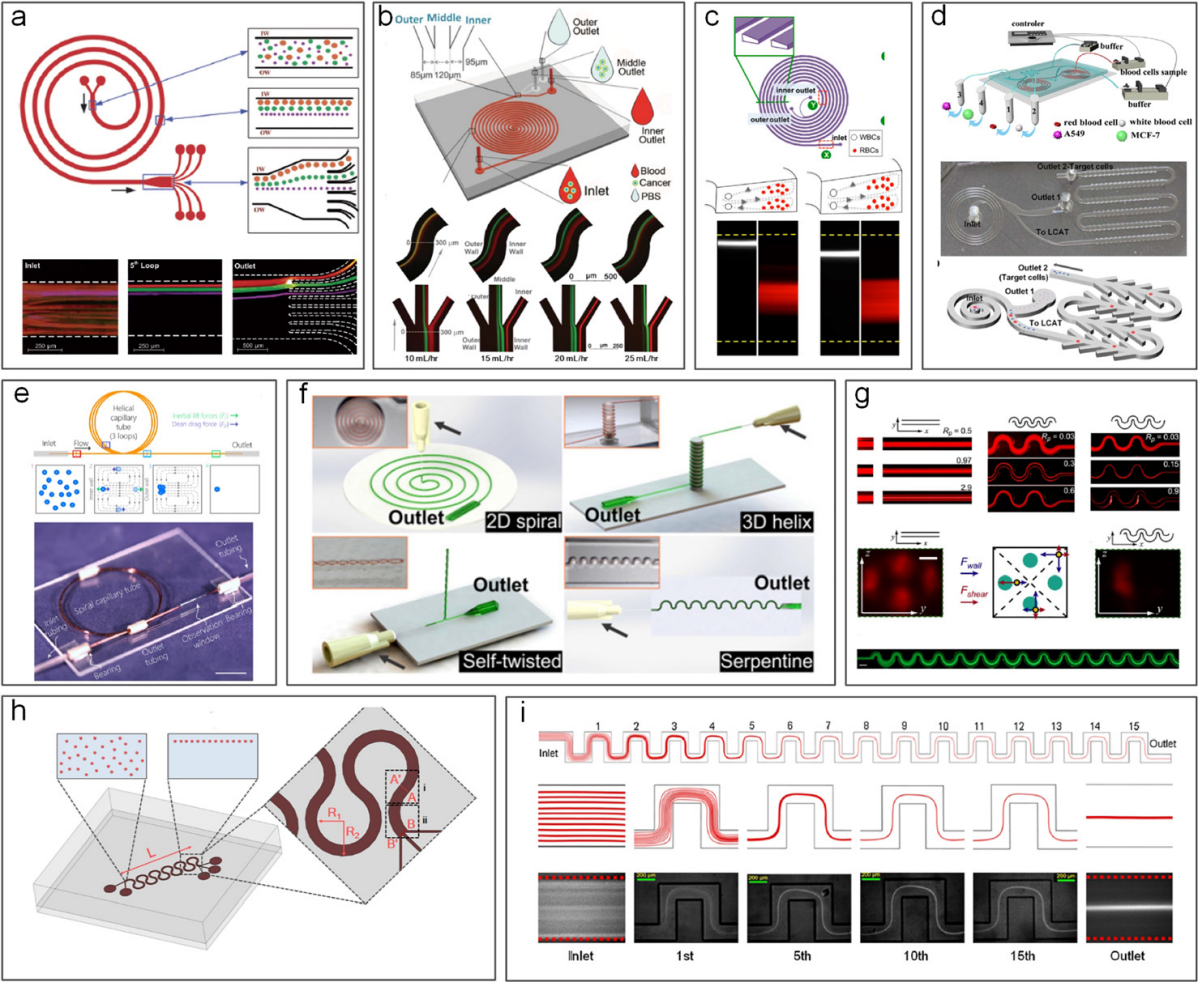
Inertial microfluidic separation using curvilinear channels. (a) Separation of 10 *μ*m, 15 *μ*m, and 20 *μ*m diameter particles in a spiral microchannel.^63^ Reproduced with permission from Kuntaegowdanahalli *et al.*, Lab on a Chip **9**(20), 2973–2980 (2009). Copyright 2009 Royal Society of Chemistry. (b) Fermat's spiral (double-spiral) channel used for tumor cell separation.^101^ Reproduced with permission from Sun *et al.*, Lab on a Chip **12**(20), 3952–3960 (2012). Copyright 2012 Royal Society of Chemistry. (c) Blood cell and particle separation in a spiral channel with a trapezoid cross section.^102^ Reproduced with permission from Wu *et al.*, Anal. Chem. **84**(21), 9324–9331 (2012). Copyright 2012 American Chemical Society. (d) A cascaded spiral microchannel^99^ and a spiral combining with an active lateral cavity acoustic transducer (LCAT) unit for blood cell separation.^95^ Reproduced with permission from Abdulla *et al.*, Anal. Chem. **90**(7), 4397–4405 (2018). Copyright 2018 American Chemical Society.^99^ Reproduced with permission from Nivedita *et al.*, Analyst **142**(14), 2558–2569 (2017). Copyright 2017 Royal Society of Chemistry.^95^ (e) A low-cost spiral channel by winding a square silica capillary into a helical form for particle focusing.^87^ Reproduced with permission from Wang *et al.*, Biomicrofluidics **11**(1), 014107 (2017). Copyright 2017 AIP Publishing. (f) Different curvilinear channels formed by winding soft Polydimethylsiloxane (PDMS) channels.^107^ Reproduced with permission from Xi *et al.*, Proc. Natl. Acad. Sci. U. S. A. **114**(40), 10590–10595 (2017). Copyright 2017 Authors licensed under a CC BY 4.0. Different forms of curved channels (g)–(i) other than spiral have also been developed for particle focusing.^61,110,111^ Reproduced with permission from Di Carlo *et al.*, Proc. Natl. Acad. Sci. U. S. A. **104**(48), 18892–18897 (2007). Copyright 2007 National Academy of Sciences. Reproduced with permission from Zhang *et al.*, Sci. Rep. **4**, 4527 (2014). Copyright 2014 Authors licensed under a CC BY 4.0. Reproduced with permission from Özbey *et al.*, Sci. Rep. **6**, 38809 (2016). Copyright 2016 Authors licensed under a CC BY 4.0.

Spiral is the most frequently used channel geometry to induce secondary Dean flows (Figs. 5 and 6). This geometry has been effectively used for micromixing applications in the past.^90^ In 2008, our group^63,89^ first demonstrated the use of spirals for focusing and sorting of microparticles and cells. We showed successful sorting of 10, 15, and 20 *μ*m diameter microparticles with an efficiency >80% in Archimedean spiral channels [Fig. 6(a)]. Similar design with low channel AR was also used by Russom *et al.*^91^ for separation of 3 and 10 *μ*m particles, where little impact of particle density (silica vs polystyrene particles) was found on the focusing behavior as suggested by the work of Yoon *et al.* using glass beads.^92^ Despite two focusing positions implied in this work,^91^ their later work^93^ reported a single focusing position similar to our early observations.^63,89^ With a redesigned spiral channel, we recently demonstrated a separation efficiency of ∼100% for particles and blood plasma at throughput up to 3 ml/min.^94^ A similar device was later integrated with an active lateral cavity acoustic transducer (LCAT) unit to achieve size-selective separation and enrichment of particles and cells^95^ [Fig. 6(d)]. The throughput of the spiral channel can be further increased by vertical stacking of a single device,^96^ and the separation performance can be improved by cascading multiple spiral devices.^97–99^

Various modifications to the spiral channel have since been proposed for enhanced particle sorting performance. Apart from the common Archimedean spiral device, Fermat's spiral [Fig. 6(b)] was also proven to be suitable for high-profile separation.^100,101^ A change to the cross-sectional geometry of the channel from the commonly used rectangular/square to trapezoidal [Fig. 6(c)] has been reported to alter the positions of the recirculating vortices for particle separation.^102–105^ The change in the focusing position of particles from the inner wall to the outer wall was observed by Guan *et al.*^103^ in their trapezoid spiral channel with a particle separation efficiency up to 92% and a flow rate up to 7.5 ml/min (Fig. 5). The shift of the focusing position at a high flow rate was also reported by Al-Halhouli *et al.*^106^ when they were separating 5 and 15 *μ*m particles. Recently, 3D spiral or helix channels were demonstrated by winding soft microtubular channels [Figs. 6(e) and 6(f)], enabling the convenient reconfiguration of microfluidic designs for particle focusing and separation.^87,107^ Additionally, multiple Dean vortices were observed in curved channels, which can also be utilized for manipulation of particle and focusing with high Dean flow (*De *>* *29).^95,108^

In addition to spiral channel geometry, serpentine channels can also be used to separate microparticles due to the presence of two cross-sectional Dean vortices.^109^ DiCarlo *et al.*^61^ used this principle to separate 10 *μ*m particles from a mixture of 10 and 2 *μ*m particles [Fig. 6(g)]. Zhang *et al.*^110^ later used a modified serpentine channel with sharp corners and achieved an efficiency > 90% in separation of similar binary mixtures [Fig. 6(i)]. Other variants of the serpentine channel were also reported for sorting particles and cytometry applications^85,111^ [Fig. 6(h)].

### C. Vortex channels

**FIG. 7.**
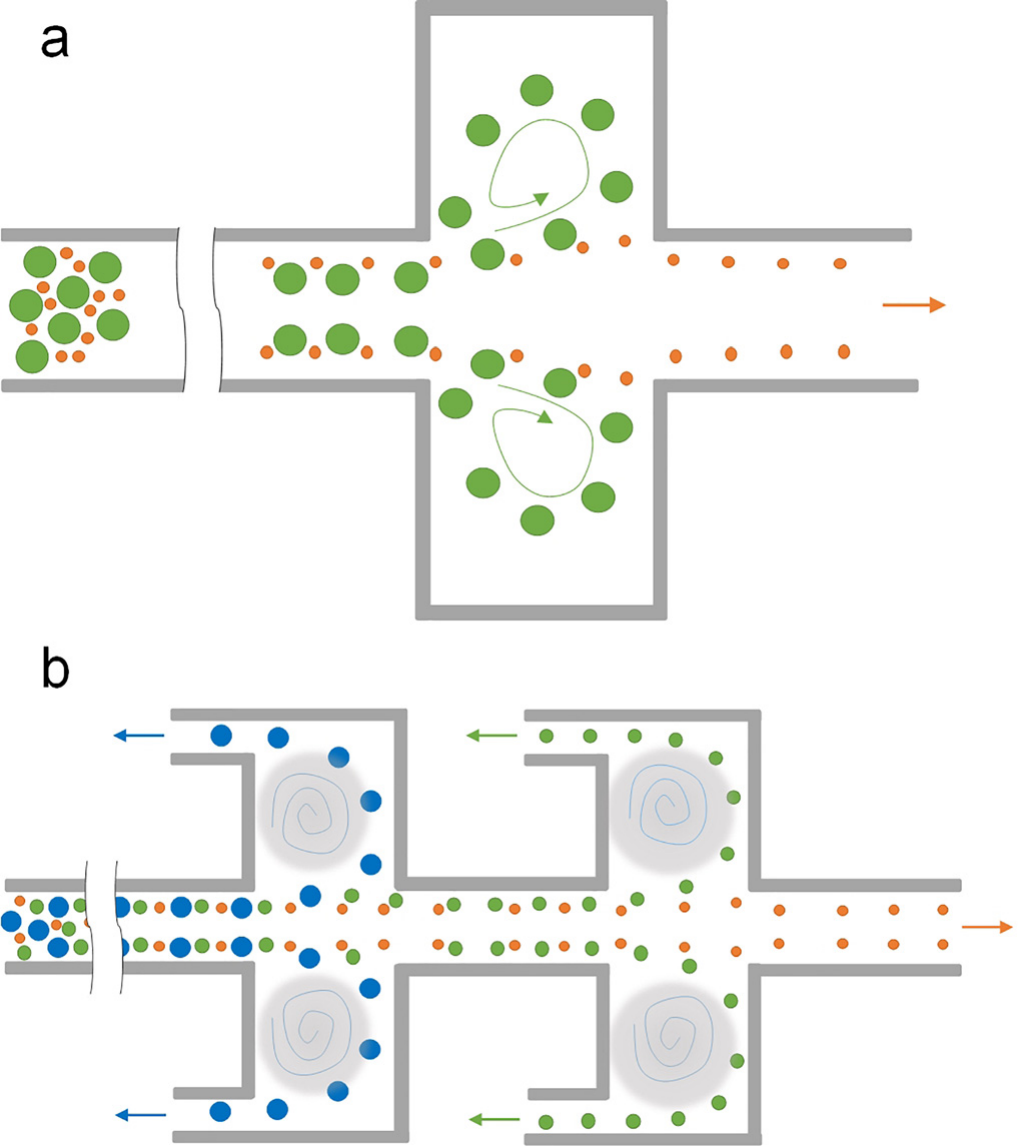
Schematic of vortex-based inertial microchannels. (a) Vortex channel by adding side chambers to a straight high AR channel for particle trapping and sorting.^114^ (b) Modified vortex channel with branch-channels attached to side chambers for continuous particle sorting.^120,121^

Counter-rotating vortices induced in spiral microchannels are continuously utilized for particle separation with high throughput (milliliter per minute). However, vortices can also be induced in channels without using the curvature (Fig. 7). While introducing the herring-bone structure on the channel roof allows to generate vortices in the channel cross-section for density-based particle separation,^112^ the use of planar or laminar vortices is more common in particle separation.

**FIG. 8.**
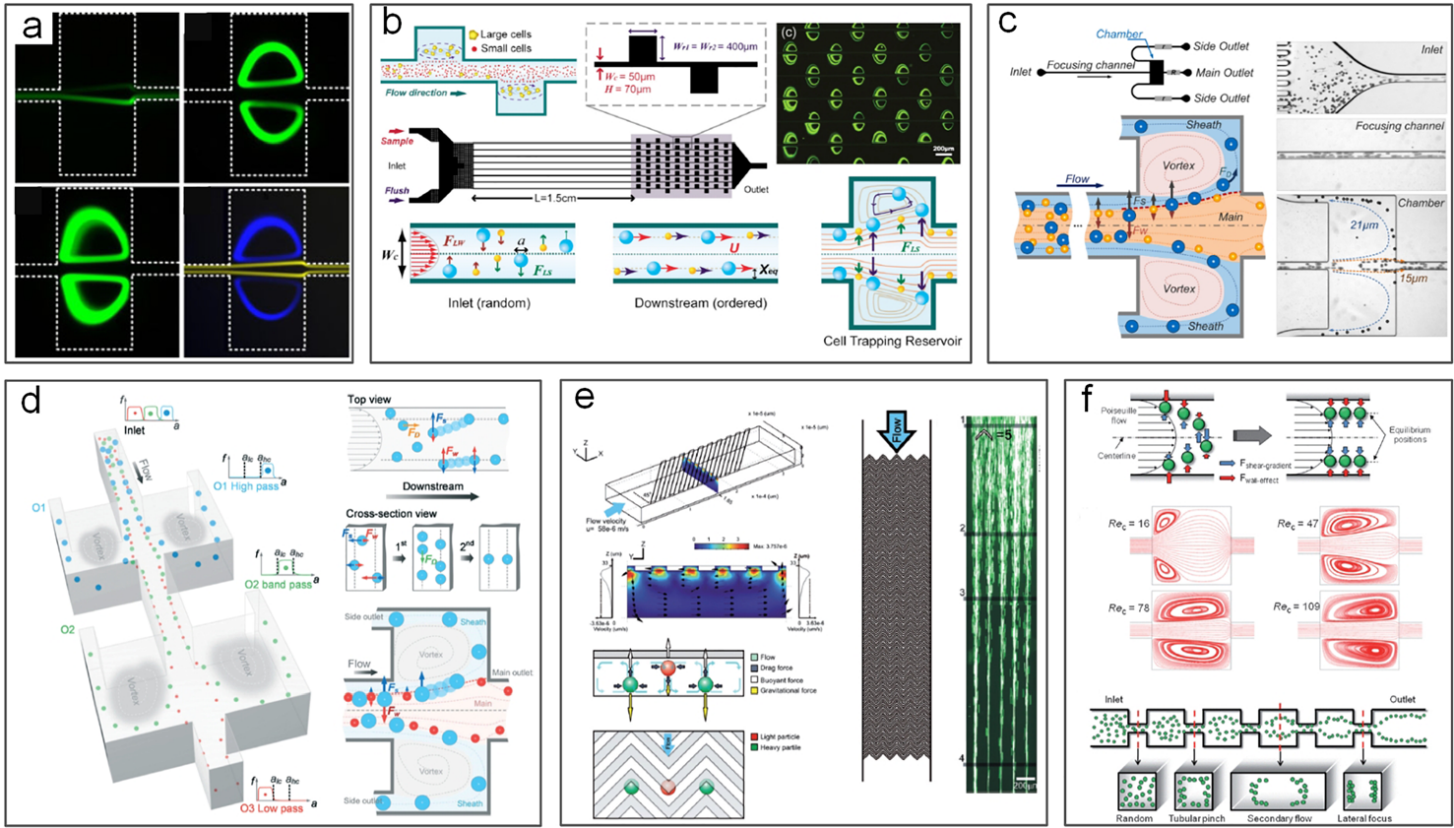
Inertial microfluidics for particle separation in vortex-based channels. (a) Separation of 15 *μ*m (yellow) and 20 *μ*m (green or blue) diameter particles in a vortex channel.^114^ Reproduced with permission from Zhou *et al.*, Microfluid. Nanofluid. **15**, 611–623 (2013). Copyright 2013 Springer Nature. (b) Multiplexed vortex-based channels for cell separation.^115^ Reproduced from Hur *et al.*, Biomicrofluidics **5**(2), 022206 (2011). Copyright 2011 AIP Publishing. (c) Modified vortex chambers with side output channels for continuous particle separation.^120^ Reproduced from Wang *et al.*, Biomicrofluidics **7**(4), 044119 (2013). Copyright 2013 AIP Publishing. (d) Cascaded side chambers for multimodal particle separation.^243^ Reproduced with permission from Wang and Papautsky, Lab on a Chip **15**(5), 1350–1359 (2015). Copyright 2015 Royal Society of Chemistry. (e) Particle separation based on density using vertical vortices generated by the grooved channel inner wall.^112^ Reproduced with permission from Hsu *et al.*, Lab on a Chip **8**(12), 2128–2134 (2008). Copyright 2008 Royal Society of Chemistry. (f) Focusing of particles in a vortex-based channel without trapping.^122^ Reproduced with permission from Park *et al.*, Lab on a Chip **9**(7), 939–948 (2009). Copyright 2009 Royal Society of Chemistry.

In 2003, laminar vortices in a microchannel were reported by Lim *et al.*^113^ when they observed recirculation of 1 *μ*m microbeads in diamond shaped microcavities attached to channel sidewalls. These vortices were due to the formation of high velocity gradients and high surface-to-volume ratios.^113^ Later, we^114^ and Di Carlo *et al.*^115^ confirmed that trapping of particles in the vortices was size-dependent and subsequently developed various microchannels with side chambers [Fig. 7(a)] to take advantage of the laminar vortices for label-free particle trapping and separation [Figs. 8(a) and 8(b)].

Selective trapping of particles in the microvortices is size-dependent due to the disruption of the balance of inertial forces in the chamber regions of the microchannel.^114^ A microchannel for particle trapping generally consists of a high AR straight segment for prefocusing particles to positions near sidewalls and a downstream segment with expansions for trapping particles into the vortices in the chambers.^114,115^ Prefocused particles experience zero net force laterally due to the balance of two inertial forces: shear-induced lift force and wall-induced lift force. However, the balance is disrupted when particles enter the expansion region with chambers where the vicinity of the channel wall suddenly disappears. As a result, wall-induced lift force is no longer present and shear-induced lift force drives particles into the side chambers where they are trapped in the vortices.^114^ Since the shear-induced lift force is strongly size-dependent, the trapping of particles is also size-selective in nature.

The throughput of vortex-based microchannels is generally very high (up to milliliter per minute) with moderate/poor efficiency and purity as large flow velocity is necessary to generate microvortices in side chambers. In our early work, a flow rate more than 300 *μ*l/min was used to isolate 20 *μ*m particles from 15 *μ*m particles.^114^ Owing to the simplicity of the channel design, the throughput can be easily scaled up by massive parallelization. Hur *et al.* showed a vortex device with 8 channels working concurrently, offering sample throughput up to 4 ml/min when capturing 10 *μ*m particles and HeLa cells.^115^ Nevertheless, the trapping efficiency of such devices is generally far from satisfactory (10%–50%).^115,116^ When biological samples are concerned, for example, isolation of rare cells,^115–119^ the efficiency can be even lower. The purity of isolated particles/cells varied from 10% to 80% depending on samples in these devices. However, due to the small volume of the chambers, their performance in terms of enrichment ratio and volume reduction rate can be very good. For example, the concentration was increased 100 000 times in our vortex-channel^114^ and an enrichment ratio up to 7 was reported.^115^

Due to the trapping mechanism, the separation in vortex-based channels is discontinuous due to the finite capacity of the side chambers for retaining particles, which in fact compromises effectively when processing large sample volumes. To overcome such limitations, we introduced a modified trapping channel [Figs. 7(b) and 8(c)],^120,121^ which included “siphoning channels” added to the side chambers to continuously extract trapped particles away and thus process samples continuously without the hassle of “flush and release.”^115–119^ Separation efficiency and purity were also enhanced in our design (both > 90%).^120^ In follow-on work, different pairs of side chambers were cascaded to demonstrate functions of low-pass, high-pass, and bandpass filters in separating particles [Figs. 7(b) and 8(d)].^121,243^ The separation performance of these devices was essentially aided by the vortices in the side chambers, despite the fact that similar channel geometries were used for separation without generating vortices^122,123^ [Fig. 8(f)].

## III. SORTING BY PINCHED FLOW FRACTIONATION

**FIG. 9.**
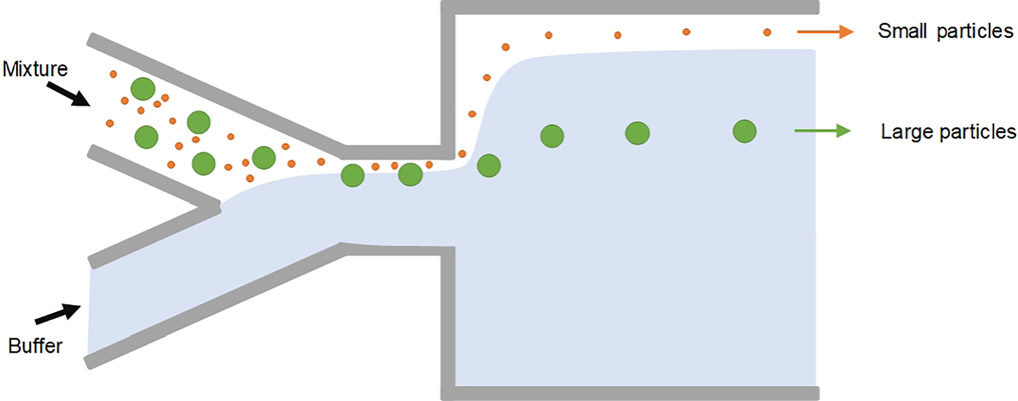
Microparticle sorting by pinched flow fractionation (PFF). Particles are pushed against the wall in the pinched segment by the buffer flow and are sorted by size in the channel broadening segment due to laminar nature of microflows.

**FIG. 10.**
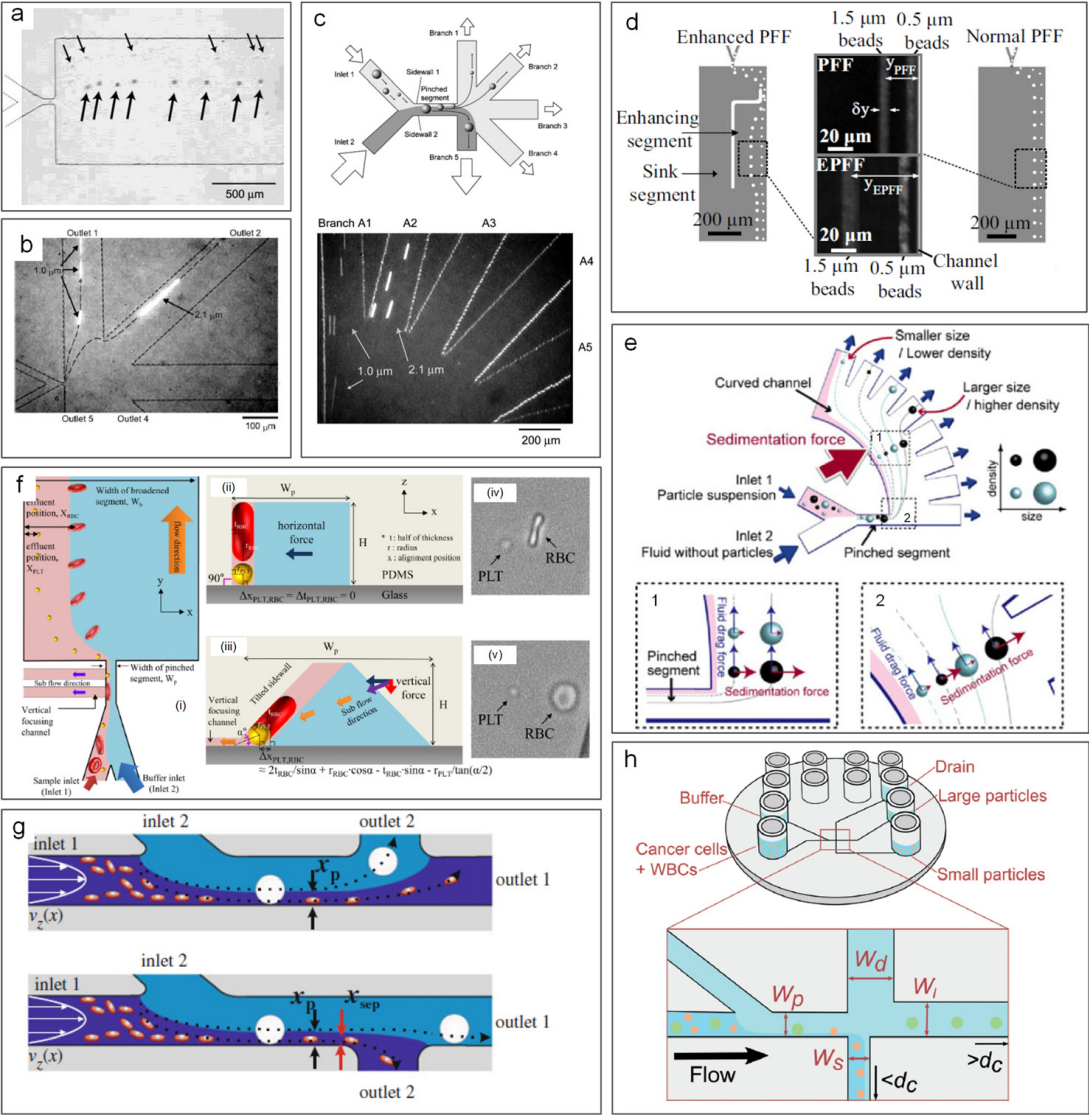
Pinched flow fractionation (PFF) for particle separation. (a) Separation of 15 *μ*m and 30 *μ*m diameter particles observed in a PFF device.^46^ Reproduced with permission from Yamada *et al.*, Anal. Chem. **76**(18), 5465–5471 (2004). Copyright 2004 American Chemical Society. (b) Separation of 1 *μ*m and 2.1 *μ*m diameter particles in a PFF channel with the PDMS membrane valve for flow control.^125^ Reproduced with permission from Sai *et al.*, J. Chromatogr. A **1127**(1-2), 214–220 (2006). Copyright 2006 Elsevier. (c) Separation in an asymmetrical PFF device.^124^ Reproduced with permission from Takagi *et al.*, Lab on a Chip **5**(7), 778–84 (2005). Copyright 2005 Royal Society of Chemistry. (d) Comparison of a normal PFF device and an enhanced PFF device.^127^ Reproduced with permission from Vig and Kristensen, Appl. Phys. Lett. **93**(20), 203507 (2008). Copyright 2008 AIP publishing. (e) Particle separation based on the size and density in a sedimentation PFF device.^133^ Reproduced with permission from Morijiri *et al.*, Microfluid. Nanofluid. **11**(1), 105–110 (2011). Copyright 2011 Springer Nature. (f) Separation of red blood cells and platelets in a PFF device with a tilted sidewall and vertical focusing channels (t-PFF-v).^130^ Reproduced with permission from Nho *et al.*, Sens. Actuators, B: Chem. **249**, 131–141 (2017). Copyright 2017 Elsevier. (g) Two channel and flow configurations for separation of leukocytes and erythrocytes.^141^ Reproduced with permission from Cupelli *et al.*, Microfluid. Nanofluid. **14**(3-4), 551–563 (2013). Copyright 2013 Springer Nature. (h) A PFF device fabricated by injection molding for cancer cell separation.^142^ Reproduced with permission from Podenphant *et al.*, Lab on a Chip **15**(24), 4598–4606 (2015). Copyright 2015 Authors, licensed under a CC BY 3.0.

Continuous sorting of microparticles based on the size can also be accomplished using pinched flow fractionation (PFF). It was first reported by Yamada *et al.*^46^ in 2004 as a passive alternative to split-flow thin (SPLITT) fractionation, which requires an external force field. The microfluidic PFF channel design is rather simple: a short (e.g., 100 *μ*m) and narrow (e.g., 50 *μ*m) microchannel as the pinched channel segment with one end having two input-branch channels and the other end with a large expansion for particle differentiation (Fig. 9). Microparticle sample flow injected into one branch channel is pinched down to a thin layer near one sidewall of the narrow channel by introducing a much faster particle-free buffer flow into the other branch channel. Due to the pinch effect, all particles are aligned to one sidewall, whereas their lateral positions are differentiated depending on their diameter, with smaller particles closer to the wall. Subsequently, the differentiated lateral positions are amplified and particles are separated when entering the expansion attributed to the laminar characteristics of microflow [Fig. 10(a)].

Many variants of the PFF approach have been proposed and demonstrated for separations based on particle physical properties including the size and shape. Following the first demonstration of PFF for size-based separation,^46^ the same group proposed an asymmetrical PFF device where the last of its five output channels was either shorter or wider (smaller flow resistance than other outputs), permitting high-resolution separation of 1 and 2 *μ*m particles^124^ [Fig. 10(c)]. With the assistance of the pressure-controlled valve at the outlet, the device can achieve a separation efficiency >97% for the same particle mixture and an efficiency of 90% for separation of 0.5 μm particles^125^ [Fig. 10(b)]. A similar device was later used for deformable droplet separation.^126^ Separation of submicron particles was also achieved in an enhanced PFF device with an embedded second stage PFF structure^127^ [Fig. 10(d)]. Using lipid vesicles with continuous size distribution, Srivastav *et al.* found a larger flow ratio of buffer and sample, which was preferred for high-quality separation, and the monodispersity quality was significantly improved in their PFF device with 30 outlets.^128^ Apart from/in addition to the flow rate ratio, the surface roughness of the channel sidewall was found to be critical for separation of small particles.^129^ Recently, a group from Korea reported separation of disk-shaped and spherical particles in their more complex version of PFF device [Fig. 10(f)] with tilted sidewalls and vertical focusing channels (termed t-PFF-v).^130^ Separation of platelets and red blood cells (RBCs) was achieved, and the separation resolution was better in the t-PFF-v device than previous classic PFF microchannels.

Apart from geometry modification, PFF can be coupled with many other effects for enhanced performance, due to its simplicity. Before PFF was demonstrated in microfluidic devices, similar concepts were already coupled with SPLITT in mesoscale devices for improved particle separation based on the size.^131,132^ Later, on the microscale, a modified PFF device was developed to combine the effect of sedimentation at the cost of complex centrifugal force fields^133^ [Fig. 10(e)]. Particles were subsequently separated efficiently according to their size and density. Similarly, the separation gap between two particles in the PFF device can be further amplified by adding a dielectrophoretic field, which was demonstrated in a recent work for the separation of 1.5 and 6 *μ*m particles.^134^ Although inertial force is usually unflavored in PFF,^46^ Lu and Xuan^135^ showed that the separation gap between particles was increased as inertia became stronger in their modified PFF device with an extensively elongated pinched segment (2 cm), and thus, better separation was achieved. Alongside the inertial effect, the same channel was used to separate 3 and 10 *μ*m particles in viscoelastic flow, where the elastic force serves to not only further enhance the separation quality^136^ but also enable the separation according to the particle shape.^137^ While most of the PFF devices employ single pinching segment, microfluidic devices with a repetitive pinching unit (also known as the contraction-expansion device) were developed to combine the effects of PFF and inertial migration for size-based separation of particles and cells.^138,139^

PFF has been widely employed for separation of various particulate samples. While most of the works in the literature are focused on separation of rigid spherical particles, size-based sorting of bubbles,^140^ droplets,^126^ and biocolloids^130^ using PFF has been reported. In recent years, separation of cells of interest has attracted increasing attention owing to its unmet need in biomedical applications.^13,14^ For example, all white blood cells (WBCs) were recovered from 10-fold diluted blood samples, while 87% of red blood cells (RBCs) were removed using a PFF device with two outlets^141^ [Fig. 10(g)]. A separation efficiency of 90% was also achieved in isolating cancer cells from WBCs using a three-outlet PFF device^142^ [Fig. 10(h)]. Overall, PFF devices show quite good separation efficiency (up to 100%^141^) and resolution (down to 1 *μ*m^124^). However, its throughput is typically lower than other separation methods, such as inertial filtration. Additionally, its requirement of high buffer flow rate is inconvenient.

## IV. SORTING BY HYDRODYNAMIC FILTRATION

**FIG. 11.**
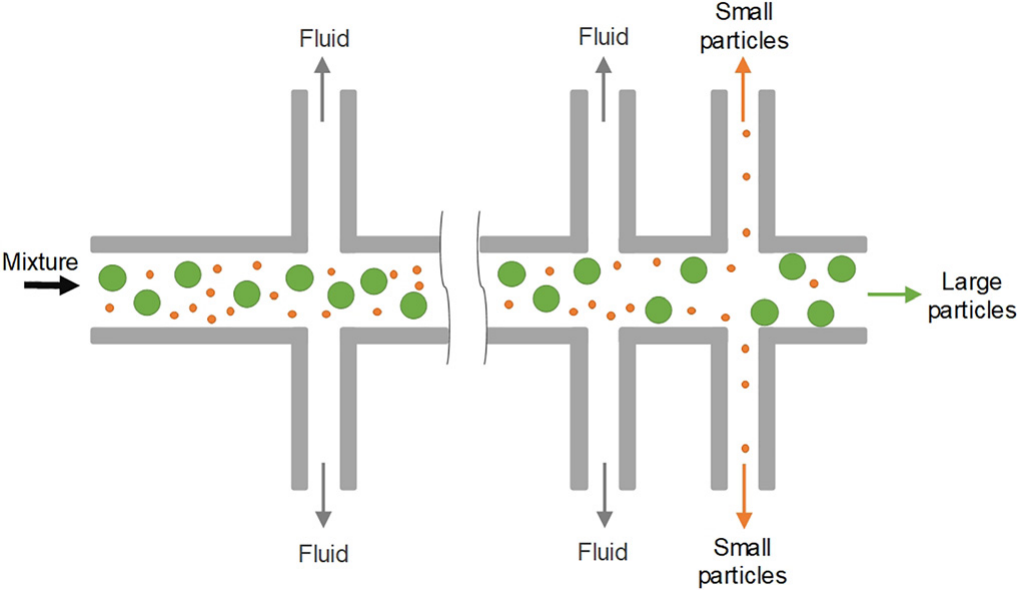
Microparticle sorting by hydrodynamic filtration (HDF). When particle suspension is introduced into the device, a small portion of the volume in the main channel is constantly siphoned into the small branch channels, leading to volume reduction and removal of particles with the size smaller than a critical diameter.

**FIG. 12.**
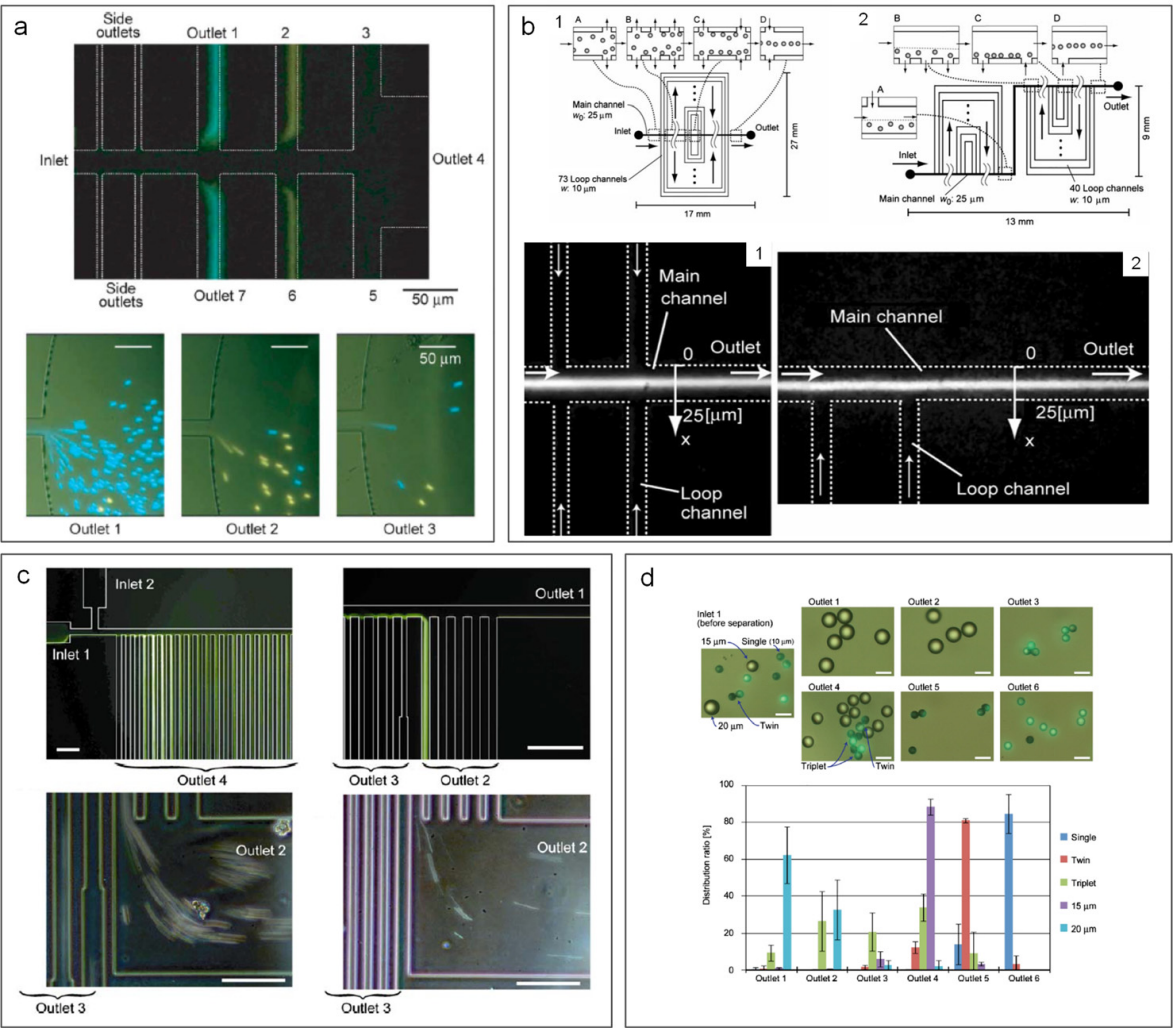
Hydrodynamic filtration (HDF) for focusing and separation of particles. (a) Separation of 2.1 *μ*m (blue) and 3 *μ*m (yellow) diameter particles in a HDF device.^47^ Reproduced with permission from Yamada and Seki, Lab on a Chip **5**(11), 1233–1239 (2005). Copyright 2005 Royal Society of Chemistry. (b) Two HDF microchannels with different side loops for sheathless hydrodynamic focusing of particles.^146^ Reproduced with permission from Aoki *et al.*, Microfluid. Nanofluid. **6**(4), 571 (2009). Copyright 2009 Springer Nature. (c) Particle and cell separation achieved in an asymmetrical HDF microchannel.^149^ Reproduced with permission from Yamada *et al.*, Biomed. Microdevices **9**(5), 637–645 (2007). Copyright 2007 Springer Nature. (d) Shape-based separation of particle singlets, doublets, and triplets using a HDF device.^150^ Reproduced with permission from Sugaya *et al.*, Biomicrofluidics **5**(2), 024103 (2011). Copyright 2011 AIP Publishing.

In 2005, Yamada and Seki^47^ pioneered a method for particle sorting, termed “hydrodynamic filtration.” Similar to pinched flow fractionation, the laminar nature of microflow was employed in this method for manipulation of particle trajectories inside a multiple-branched microchannel [Figs. 11 and 12(a)]. When a particle suspension was introduced into their device, a small portion of the volume in the main channel was constantly siphoned into the 50 small branch channels, leading to the volume reduction and removal of particles with the size smaller than a critical diameter. Particles larger than this diameter were excluded from entering the branch channels due to the presence of the channel wall. Despite the seeming similarity of this method to conventional membrane filtration, the working mechanisms and key dimensions are different. The sizes of side branch channels (5 × 5 *μ*m^2^) in hydrodynamic filtration are larger than particle sizes (1–3 *μ*m), while the pore size of the conventional filtration membrane must be smaller than that of the largest particles in the suspension. A recovery rate of ∼60% was achieved for separation of 3 *μ*m particles from a mixture of 1, 2, and 3 *μ*m particles in the pioneering work.^47^

Since the seminal work,^47^ modified hydrodynamic filtration devices have been proposed for focusing and separation of particles and cells (Fig. 12). Hydrodynamic focusing^143^ is the key element of the widely used benchtop flow cytometry^144^ and its microscale counterparts,^145^ where sheath flow is required to focus sample flow into a thin stream for downstream analysis. In 2009, Aoki *et al.* ingeniously achieved hydrodynamic focusing without additional sheath flow using a hydrodynamic filtration device^146^ [Fig. 12(b)]. In their modified device, side channels were looped back to the main channel. Thus, the particle-free liquid, which was drawn from upstream of the main channel, was repurposed as sheath flow when it flowed back into downstream of the main channel, leading to successful hydrodynamic focusing of particles at the end of the channel. ∼100% focusing of 5 *μ*m particles was achieved in their channel without external sheath flow. Nevertheless, the same group later introduced an external sheath flow into an asymmetrical hydrodynamic filtration device where subpopulations of leukocytes were separated based on the size.^147^ Similar designs were also used for size-based cell-cycle synchronization,^148^ shape-based differentiation of single and clustered cells^149,150^ [Figs. 12(c) and 12(d)], and deformability-based sorting of droplets.^151^ With the critical diameter set to 320 nm, Fouet *et al.* showed extraction of 100 nm beads from a complex mixture of particles.^152^

Hydrodynamic filtration has proven to be an excellent approach for manipulation and separation of small particles inside a membraneless microchannel. It has been used for sorting near-micro^47^ and submicroparticles^152^ as well as cell classifications.^147^ While the resolution is quite high and the device is flexible, as it can be easily modified for sorting different sample, its separation performance in terms of efficiency and purity remains to be improved. Furthermore, in order to take advantage of laminar flow, the throughput of such a system is generally limited (e.g., 1–25 *μ*l/min^47,147^) to avoid the potential influence of inertia, and thus, it is not suitable for processing a large volume of sample. The employment of tens of side channels also complicates channel design and practical operation.

## V. SORTING BY CROSS-FLOW FILTRATION

**FIG. 13.**
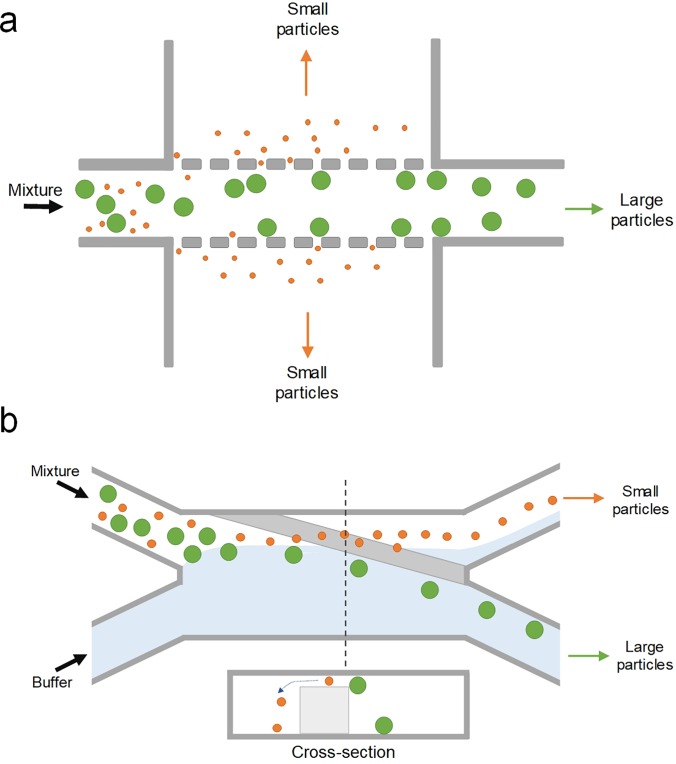
Microparticle sorting by cross-flow filtration (CFF). Sample solution flows tangentially across the filter structure in a CFF device, and the permeable solution flows laterally rather than vertically through the membrane. Particles with sizes smaller than the pores or gaps on the filter pass through the filter, and all other larger ones are washed away. The filter structure is typically micropostlike (a), but slanted weir structure (b) can also be used.

Cross-flow filtration (CFF) is one of the microfiltration methods that separate particles mainly based on their size by transmembrane pressure.^48^ Unlike the conventional filtration using dead-end filters, sample solution flows tangentially across the membrane in a cross-flow filtration device and the permeable solution flows laterally rather than orthogonally through the membrane (Fig. 13).^153^ With transmembrane pressure, particles with sizes smaller than the pores or gaps on the filter pass through the filter, while all others are washed away,^154^ effectively eliminating clogging issues commonly observed in dead-end filters.^155^ This approach extends the performance range of microfluidic sorters into the 100 nm range, with throughputs as high as 100 *μ*l/min (Fig. 1).

**FIG. 14.**
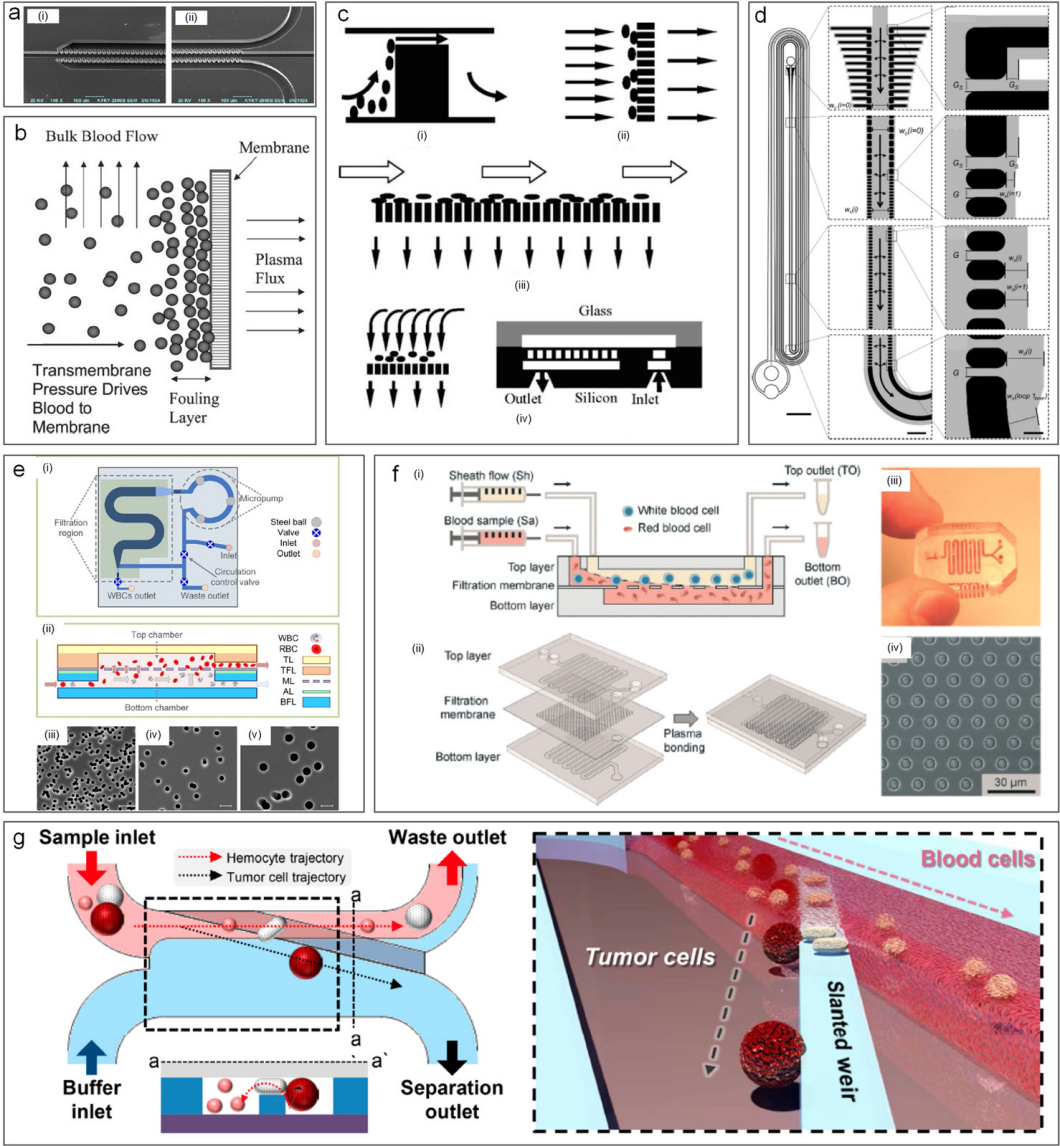
Cross-flow filtration used for particle separation. (a) SEM image of a cross-flow microfluidic channel using pillars for blood separation.^163^ Reproduced with permission from Chen *et al.*, Sens. Actuators, B: Chem. **130**(1), 216–221 (2008). Copyright 2008 Elsevier. (b) Porous membrane used in a cross-flow device for blood plasma filtration.^168^ Reproduced with permission from Crowley and Pizziconi, Lab on a Chip **5**(9), 922–929 (2005). Copyright 2005 Royal Society of Chemistry. (c) Comparison of weir filter, dead-end pillar filter, cross-flow filtration, and membrane filter for blood separation.^160^ Reproduced with permission from Ji *et al.*, Biomed. Microdevices **10**(2), 251–257 (2008). Copyright 2008 Springer Nature. (d) A cross-flow looped device for leukocyte reduction in plasma.^244^ Reproduced with permission from Xia *et al.*, Sci. Rep. **6**, 35943 (2016). Copyright 2016 Authors, licensed under a CC BY 4.0. (e) Porous membrane based cross-flow devices for discontinuous leukocyte separation.^166^ Reproduced from Cheng *et al.*, Biomicrofluidics **10**(1), 014118 (2016). Copyright 2016 AIP Publishing. (f) Continuous separation using the porous membrane.^159^ Reproduced with permission from Li *et al.*, Lab on a Chip **14**(14), 2565–2575 (2014). Copyright 2014 Royal Society of Chemistry. (g) Slanted weir based microchannel for separation of circulating tumor cells from blood.^161^ Reproduced with permission from Yoon *et al.*, Cancers **11**(2), 200 (2019). Copyright 2019 Authors licensed under a CC BY 2.0.

The CFF devices can be roughly categorized based on filter designs including membrane filter, pillar filter [Fig. 13(a)], and weir filter [Fig. 13(b)].^153^ Membranes filters can be made of various materials, and their geometry is quite versatile, such as flat, tubular, multitubular, hollow-fiber, capillaries, or spiral-wound.^156^ Cross-flow devices using these membrane filters offer extended fields of applications in industrial processes,^157^ from pharmaceutical fractionation^158^ to blood preprocessing^159^ (Fig. 14). Pillar filters consist of rows of pillarlike cylinders with critical cut-off dimensions.^160^ Higher flow velocity and more uniform flow profiles were observed in cross-flow devices with slanted pillar filter^160^ despite the fact that black flow issues may occur in such devices.^20^ The last group of devices with weir filter features long microbarriers to only allow small particles to go through.^56,161^ Such devices overcome the backflow issues^20^ with trade-off of potentially decreased separation efficiency.^160^

Cross-flow microfiltration has a wide range of applications, such as separation on nano-^162^ and microscales,^163^ enrichment,^244^ and isolation of extracellular vesicles^164^ and CTCs^161^ from complex biosamples. Yoon *et al.*^161^ utilized weir filters to continuously separate CTCs from whole blood, achieving a separation efficiency of 97% [Fig. 14(g)]. They took account of the size and deformability of CTCs (300–350 *μ*m^2^). Similarly, Chen *et al.*^163^ developed an integrated device for cell separation, cell lysis, and DNA purification. 91.2% RBCs were removed by the weir-type chip with the gap of 3.5 um from a diluted blood.^163^ Ji *et al.* compared four types of cross-flow devices and concluded that pillar-type microfilters were best for on-chip genomic analysis^160^ [Fig. 14(c)]. Generally, lower permeation efficiency is inevitable with the reducing transmembrane pressure caused by the increasing permeate viscosity during the process. Fortunately, this issue can be mitigated by widening side channels gradually. Gifford *et al.*^165^ developed a pillar-type incremental filtration device that precisely controlled the amount of fluid diverted at each filtration gap. It separated 1 *μ*m particles at a flow rate of 500 *μ*l/min and achieved an ∼3× enrichment of platelets with 80%–85% yield.^165^ In addition to the pillars and weirs, membrane microfilters are also used widely in filtration. Cheng *et al.*^166^ reported a hybrid device with a microporous membrane (3 *μ*m pore size) for separating binary microbeads and WBCs from whole blood [Fig. 14(e)]. This device integrates both dead-end and cross-flow filters with a bidirectional micropump, offering a recovery rate of ∼72% at a throughput of ∼38 *μ*l/min.^166^

So far, the main application of cross-flow filtration is sample pretreatment of whole blood, including plasma,^167–169^ RBC/WBC,^20,160,163,167,170^ and CTC separations.^161^ Despite high-throughput and clogging-free separation, particle attachment to the filters can induce the issue of channel blocking.^48^ Additionally, deformation of the particles can affect the performance of microfilters.^171^ Although such a disadvantage can be used for cell sorting by stiffness,^172^ this limitation needs to be considered during device development.

## VI. SORTING BY DETERMINISTIC LATERAL MIGRATION

**FIG. 15.**
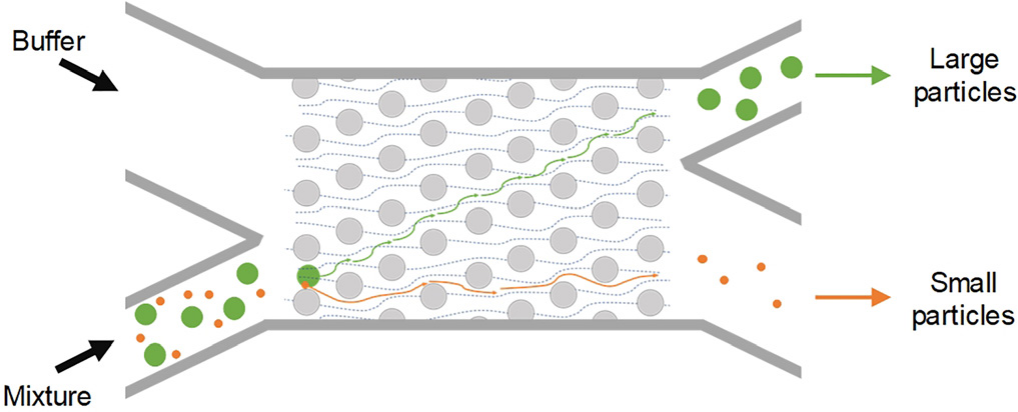
Microparticle sorting by deterministic lateral migration (DLD). Each row of the posts in the array is laterally shifted from the preceding row by a certain distance, continuously dividing the flow inside the DLD device to create separate streamlines. Under laminar flow conditions, particles smaller than the critical size follow their initial streamline in the flow, while those larger are displaced into adjacent streamlines (dashed lines in the FIG) due to particle-micropost interactions.

Deterministic lateral displacement (DLD) is another size-based method for continuous particle separation. It has been extensively investigated and widely adopted for various applications since its first demonstration by Huang *et al.* in 2004.^49,173^ It is compatible with a similarly wide, 3-orders of magnitude range of particle sizes as inertial microfluidics discussed earlier (from tens of nanometers to tens of micrometers), while offering the broadest range of throughput from nanoliter per minute to microliter per minute (Fig. 1). The key functional component of the DLD device is its carefully arranged postarray, where each row of the posts is laterally shifted from its preceding row by a certain distance (Fig. 15). The misaligned posts continuously divide the flow inside the DLD device and create separate streamlines. Under laminar flow conditions, particles smaller than a critical size follow their initial streamline in the flow and those larger are displaced into adjacent streamlines due to particle-micropost interaction. As a result, particles can be conveniently separated based on their size.

**FIG. 16.**
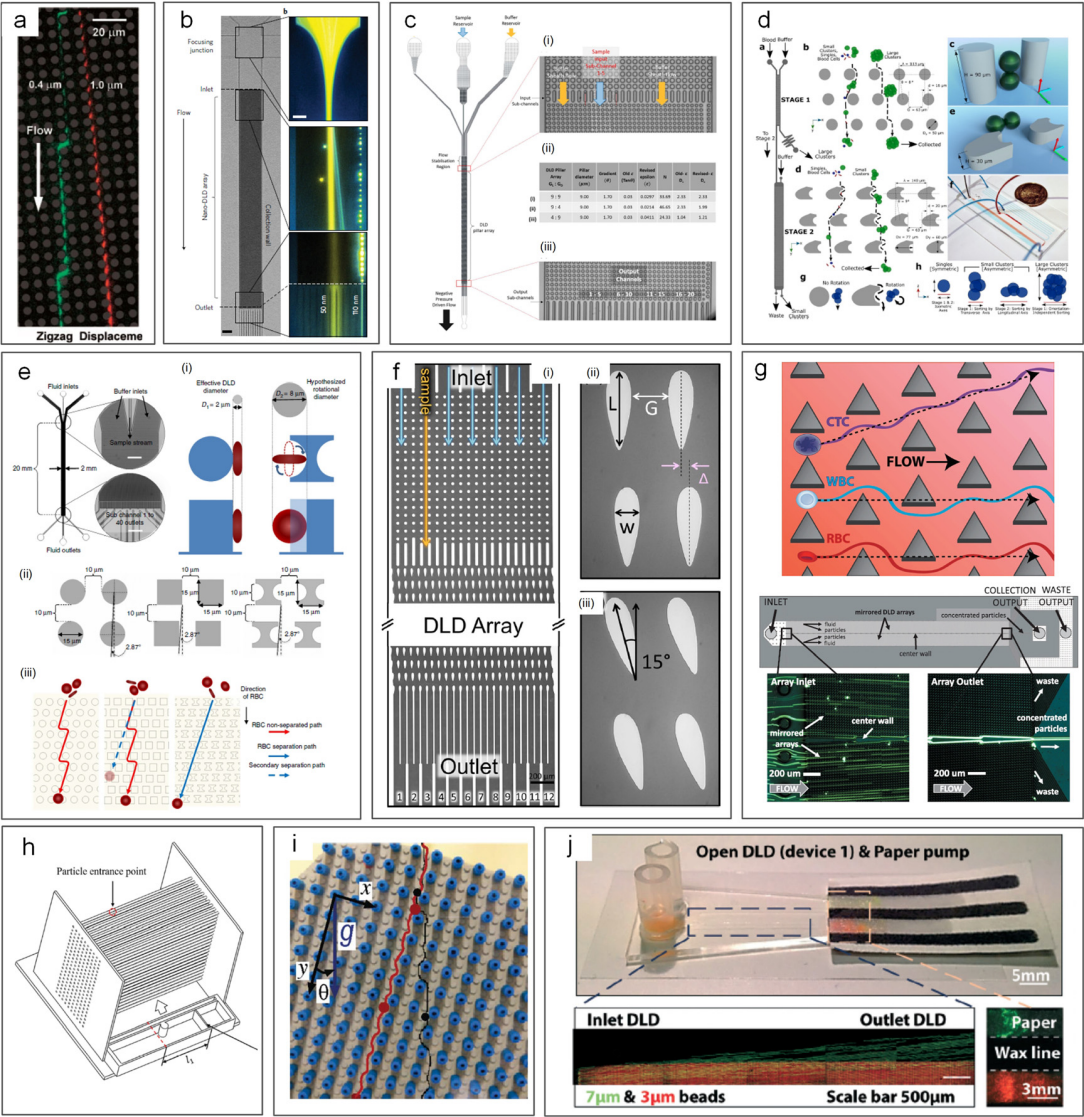
Deterministic lateral displacement (DLD) for particle separation. (a) A DLD device first reported, capable of submicrometer particle separation.^49^ Reproduced with permission from Huang *et al.*, Science **304**(5673), 987–990 (2004). Copyright 2004 American Association for the Advancement of Science. (b) Separation of 50 and 100 nm diameter particles in a nano-DLD device.^179^ Reproduced with permission from Wunsch *et al.*, Nat. Nanotechnol. **11**, 936–940 (2016). Copyright 2016 Springer Nature. (c) DLD device using different postarray gaps.^175^ Reproduced with permission from Zeming *et al.*, Sci. Rep. **6**, 22934 (2016). Copyright 2016 Authors licensed under a CC BY 4.0. (d) DLD device using asymmetrical pillar arrays.^184^ Reproduced with permission from Au *et al.*, Sci. Rep. **7**(1), 2433 (2017). Copyright 2017 Authors licensed under a CC BY 4.0. (e) DLD device using square and I-shaped pillar arrays.^174^ Reproduced with permission from Zeming *et al.*, Nat. Commun. **4**, 1625 (2013). Copyright 2013 Springer Nature. (f) DLD device using airfoil pillar arrays.^176^ Reproduced with permission from Dincau *et al.*, Microfluid. Nanofluid. **22**(12), 137, (2018). Copyright 2018 Springer Nature. (g) DLD device using triangular pillar arrays.^181^ Reproduced with permission from Loutherback *et al.*, AIP Adv. **2**(4), 042107 (2012). Copyright 2017 Authors licensed under a CC BY 3.0. (h) A 3D-DLD device used for particle separation driven by gravity.^183^ Reproduced with permission from Du and Drazer, Sci. Rep. **6**, 31428 (2016). Copyright 2016 Authors licensed under a CC BY 4.0. (i) A force-driven DLD array made from LEGO board and pegs for water droplet separation.^182^ Reproduced with permission from Drazer *et al.*, Lab on a Chip **12**(16), 2903–2908 (2012). Copyright 2012 Royal Society of Chemistry. (j) An open-DLD device driven by the paper pump for bead separation.^245^ Reproduced with permission from Tran *et al.*, Lab on a Chip **17**, 3592–3600 (2017). Copyright 2017 Authors licensed under a CC BY 3.0.

Various modifications from original DLD design have been reported for improved performance in terms of less clogging, hydrostatic pressure requirements, and displacement characteristic range. Apart from the typical circular posts used by Huang *et al.*^49^ [Fig. 16(a)], a variety of micropost geometries have been reported [Figs. 16(c)–16(g)]. These include triangular I-shaped [Fig. 16(e)],^174,175^ rectangular,^49,173^ and airfoil-shaped posts [Fig. 16(f)].^176^ By adopting triangular micropost arrays [Fig. 16(g)], clogging issues in the DLD device were significantly mitigated as a result of the larger working gap size between triangular microposts. Zeming *et al.*^174,175^ developed a DLD device with I-shaped pillars, permitting effective separation of nonspherical bioparticles undergoing rotational movements. In their work, RBCs were effectively filtered from the blood sample. To avoid generation of vortex and compressed streamlines, low “Reflow” and thus low throughput are generally necessary.^177^ Nevertheless, a much higher throughput has been achieved in a DLD device with symmetric airfoil pillars in a recent work.^176^ Vortices were not observed at *Re* up to 100, and 20 *μ*m diameter particles were successfully separated from 10 *μ*m and 15 *μ*m particle mixtures with an efficiency of ∼100%.^176^

DLD devices can be adapted for separation of particles with a wide size range, from the millimeter-scale^178^ down to tens of nanometers.^179^ Inglis *et al.* demonstrated a 99% recovery for separating 4.2 *μ*m particles from 2.1 *μ*m and 5.7 *μ*m particles using the slightly tilted column of circular posts. With a shift fraction of only 0.006 and a 16.5 *μ*m gap, clogging was not observed in their design.^180^ Similarly, triangular posts show fewer clogging issues. Loutherback *et al.* used triangular posts to successfully separate 15–30 *μ*m large CTCs from blood with a recovery rate of 85% and a flow rate up to 10 ml/min.^181^ While most of the DLD devices were developed for microparticle separation, DLD can also be tuned for macroscale^182^ and nanoscale^179^ separations. In 2012, water drops with the diameter ranging from 3.7 mm to 10.2 mm were successfully separated in a gravity-driven DLD device made from LEGO® pegs and boards [Fig. 16(i)].^182^ The same group later extruded their 2D device into a 3D gravity-driven DLD [Fig. 16(h)] for separating 3.16 mm particles from smaller particles (1.59 and 2.38 mm) with an efficiency of 100%.^183^ On the other hand, Wunsch *et al.*^179^ demonstrated a nano-DLD array with a 25 nm gap [Fig. 16(b)] for fractionating colloids with diameters down to 20 nm even at *Pe *≥* *4. The same group built a phenomenological model to analyze the size separation cutoff qualitatively by controlling the gap size, flow velocity, and length of arrays. Their design recovered over 75% of the 2.0 kb DNA fragment and threefold concentration from *HindIII* digested lambda phage DNA with a gap size of 238 nm at ∼200 *μ*m/s.^19^

In general, DLD is a flexible and versatile method that can easily be modified for various applications despite some limitations. Since most bioparticles (e.g., cells) are a few micrometers in diameter, DLD devices have been widely used in biomedical applications such as isolation of CTCs from blood,^181,184,185^ separation of WBCs and RBCs,^175,186^ separation of exosomes and colloids,^179^ and isolating parasites in microfluidics.^187,188^ Even a paper microfluidic based design has been demonstrated^245^ [Fig. 16(j)]. Nevertheless, DLD is not without limitations, with diffusion and fluidic resistance being its two main challenges.^173,189^ Diffusion in DLD devices is generally unfavored, as the random Brownian motion of particles disturbs the otherwise static laminar flow and tends to cause particle mixing, leading to downgrading separation performance especially for submicrometer particles.^155^ Similarly, for separation of submicrometer particles, the gap between microposts has to be reduced accordingly, causing significantly increased fluid resistance.^190^ Excessive resistance requires extremely large pressure, which may not be easily accessible.^191^ Additionally, the exceedingly large surface area to volume ratio due to numerous micro/nanoposts may result in particle binding to the channel surface and thus device clogging.^173^

## VII. GRAVITY-BASED SORTING

**FIG. 17.**
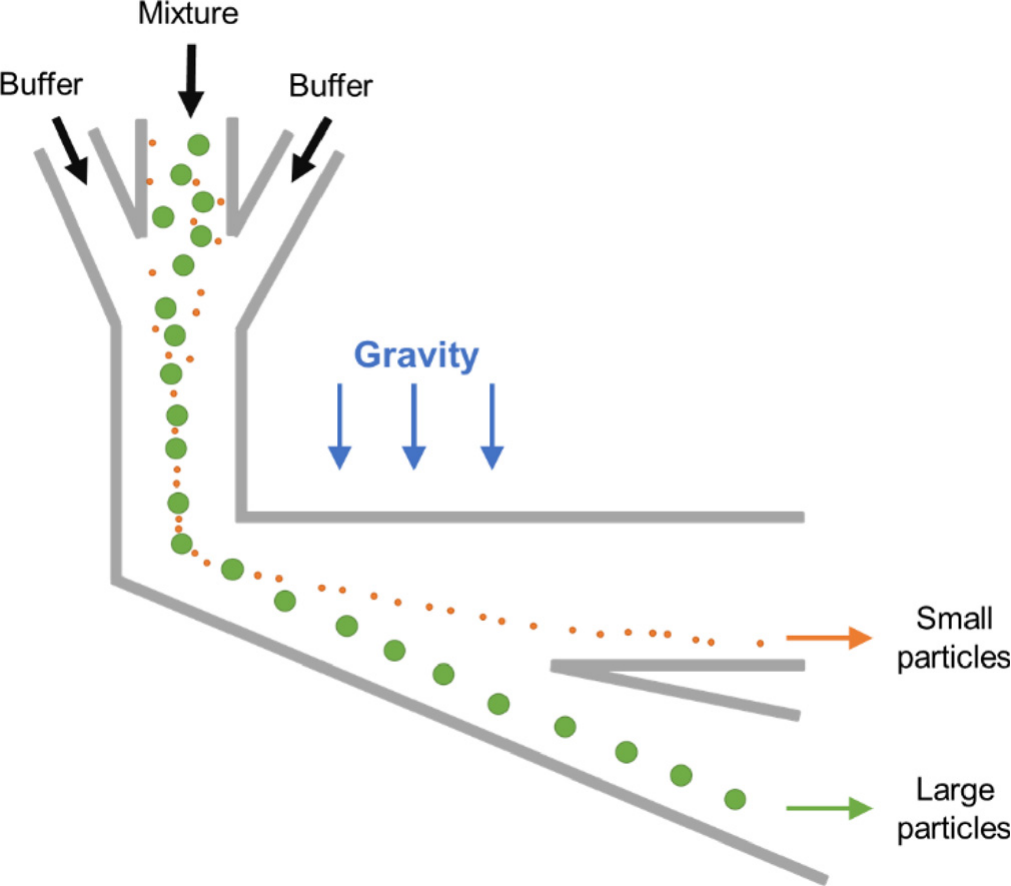
A representative design of gravity-based sorting. Particle mixture is first focused into a thin stream by hydrodynamic focusing in a vertical channel segment. Thereafter, once the focused particles reach the horizontal channel with vertical expansion, they are separated by particle mass/size undergoing the sedimentation effect.

Gravity is frequently incorporated into microfluidic devices for particle separation due to its ubiquitous presence and coupling simplicity.^10^ Since no artificial force field and its control units are required, we also discuss separation microsystems taking advantage of gravity (Fig. 17). These systems might be deemed as quasipassive label-free separation technologies, which are based on particle properties such as density. Among these technologies are centrifugation,^192^ field-flow fractionation (FFF),^10^ and split-flow thin (SPLITT) fractionation.^193^ Various and efficient microsystems of these kinds have emerged for separating particles with different masses and sizes utilizing natural or artificial gravity.^194^

**FIG. 18.**
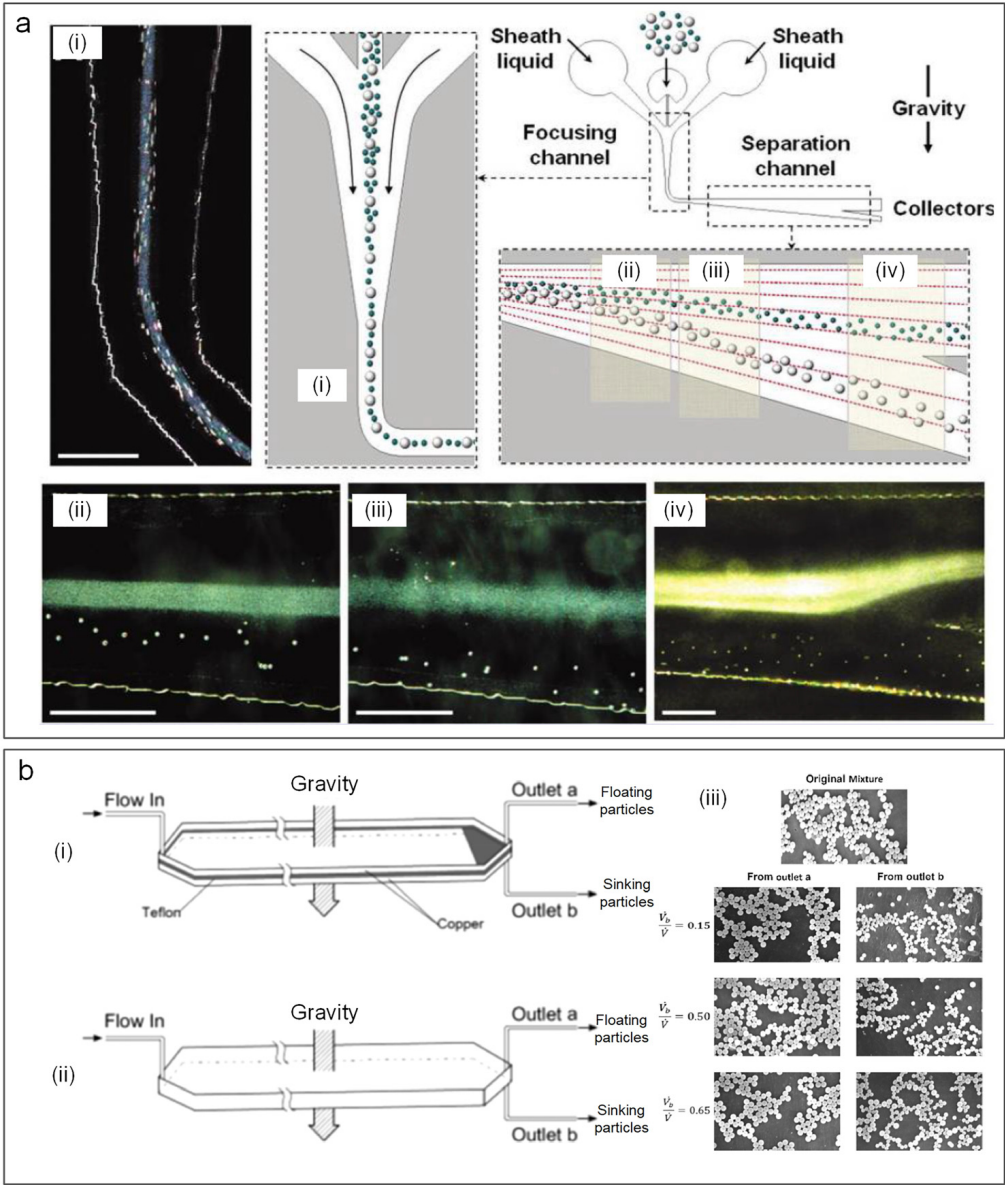
Separation of particles involving natural gravity. (a) Particle separation based on the mass and size in a microchannel with the hydrodynamic prefocusing segment and an expanding segment taking advantage of natural gravity.^199^ Reproduced with permission from Huh *et al.*, Anal. Chem. **79**(4), 1369–1376 (2007). Copyright 2007 American Chemical Society. (b) Sorting of light and heavy particles in split-flow thin (SPLITT) channels in the gravity field.^193^ Reproduced with permission from Barman *et al.*, Ind. Eng. Chem. Res. **57**(6), 2267–2276 (2018). Copyright 2018 American Chemical Society.

These gravity-based systems are popular for their high separation performance. Owing to their controllability over artificial gravity magnitude, numerous centrifugal microfluidic platforms have been proposed^195^ by using rotating disks. Most of them are relevant to blood sample preparation,^196^ cell-based assays,^197^ and DNA extraction.^198^ On the other hand, natural gravity-driven microfluidic systems can also deliver high separation efficiency with specified channel designs. Huh *et al.*^199^ reported a device with hydrodynamic separation amplification [Fig. 18(a)]. The key of this device is a flow pattern where fluid streams spread progressively into the widening of the separation channel, leading to reduced flow velocity. As a result, more time is available for gravity to take effect and cause sedimentation of particles. This method can separate particles that are larger than a diameter of 6 μm from the smaller ones with 99.9% high-purity at a flow rate of 1 ml/h.^199^

Coupling of gravity force with other separation techniques like PFF, FFF, and SPLITT offers effective separation of particles. In 2011, Morijiri *et al.*^133^ presented microfluidic systems combining size-based sorting technique PFF with centrifugal microfluidics, achieving separation of particles with different densities (1.05 g/cm^3^ and 2 g/cm^3^) and sizes (3 and 5 *μ*m). Barman *et al.*^193^ continuously and rapidly separated particles of two different densities by employing the sink-float phenomenon in split-flow thin (SPLITT) cells using both centrifugal and gravitational fields [Fig. 18(b)]. Their separation efficiencies were 60%–98% for 0.586, 0.822, 5, and 15 *μ*m beads.^193^

Natural and artificial gravity-driven separation methods are flexible and easy to be coupled with other functional microfluidic components, permitting high resolution, pump-free separation within a closed fluidic system.^200^ As a result, these separations have been widely used for *in vitro* diagnostic testing at the point-of-care.^10^ However, it has a few drawbacks as well. Due to the diffusion and fluid dynamic Rayleigh-Taylor-like instability,^194^ small particles are always in the trend of mixing and chaos, which is unfavored and can be mitigated by using a density gradient to counteract the instabilities and inert molecules. More importantly, artificial gravity-driven systems do not require a continuous separation method,^192^ which extensively limits their throughput.

## VIII. VISCOELASTIC SEPARATION

**FIG. 19.**
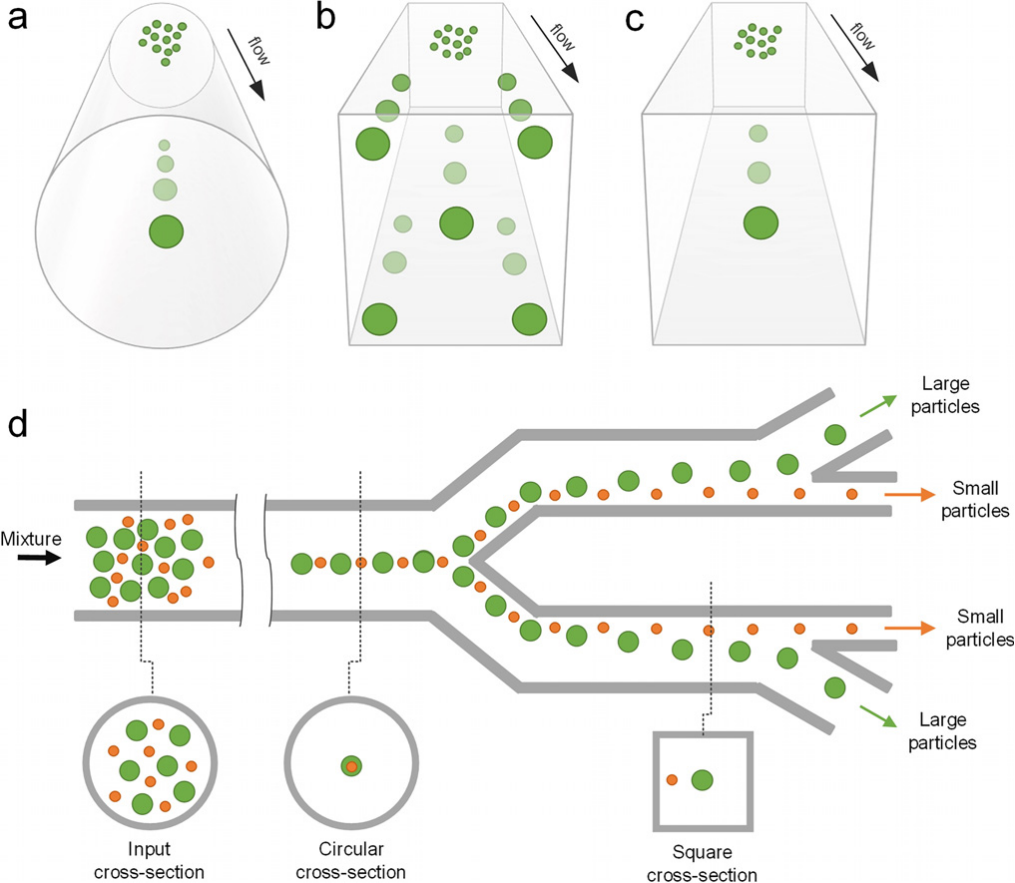
Viscoelastic focusing and sorting of particles. (a) Viscoelastic focusing creates a single equilibrium position along the central axis of a circular capillary. In square channels, there are (b) five focusing positions when flow is elasticity dominant and (c) a single position at the centerline when flow is elastoinertial. (d) Size-based sorting of particles using elasticity dominant flow in a device with a combination of circular and square cross sections.

Microparticle sorting methods discussed so far are based on manipulating hydrodynamic forces in Newtonian fluids. However, biological fluids, such as blood,^57^ saliva,^201^ and cytoplasm,^202^ are non-Newtonian fluids, which can impact the effectiveness of the aforementioned methods for particle separation. These biofluids are generally viscoelastic in nature, making separation of particulate elements within them challenging.^57^ Fortunately, the fluid viscoelasticity offers unique opportunities to focus particles into different cross-sectional locations in a microchannel, depending on their size as particles suspended in such flows are subjected to an elastic lift force (Fig. 19).^203,204^ Such viscoelastic focusing of particles is especially advantageous in forming a single-stream 3D-focusing in square microchannels^203,205^ and in entrainment of submicrometer particles,^206,207^ which is generally challenging in other microfluidic systems such as inertial microfluidic devices (Fig. 1).

Typical macromolecules used for enhancing fluid elasticity include hyaluronic acid (HA),^208^ poly(ethylene oxide) (PEO),^209^ deoxyribonucleic acid (DNA),^210^ and poly(vinylpyrrolidone) (PVP).^211^ The rheological properties of such viscoelastic flow can be assessed by the nondimensional Weissenberg number (*Wi* = *λ*$\dot{\gamma }$, where *λ* is the fluid relaxation time and $\dot{\gamma }$ is the shear rate), which compares elastic force with viscous force acting on suspended particles.^205^ For a rectangular channel, *Wi* can be expressed as $Wi=\frac{2Q\lambda }{wh^{2}}$, where *Q* is the flow rate. Another useful parameter is the elasticity number (*El*),^212^ which indicates the relative importance of elastic and inertial forces in a shear flow.^213^ For a rectangular channel, $El=\frac{Wi}{Re}=\frac{\lambda \mu (h+w)}{\rho h^{2}w}$ and is independent of flow conditions with constant viscosity since both *Wi* and *Re* are proportional to the flow rate.

**FIG. 20.**
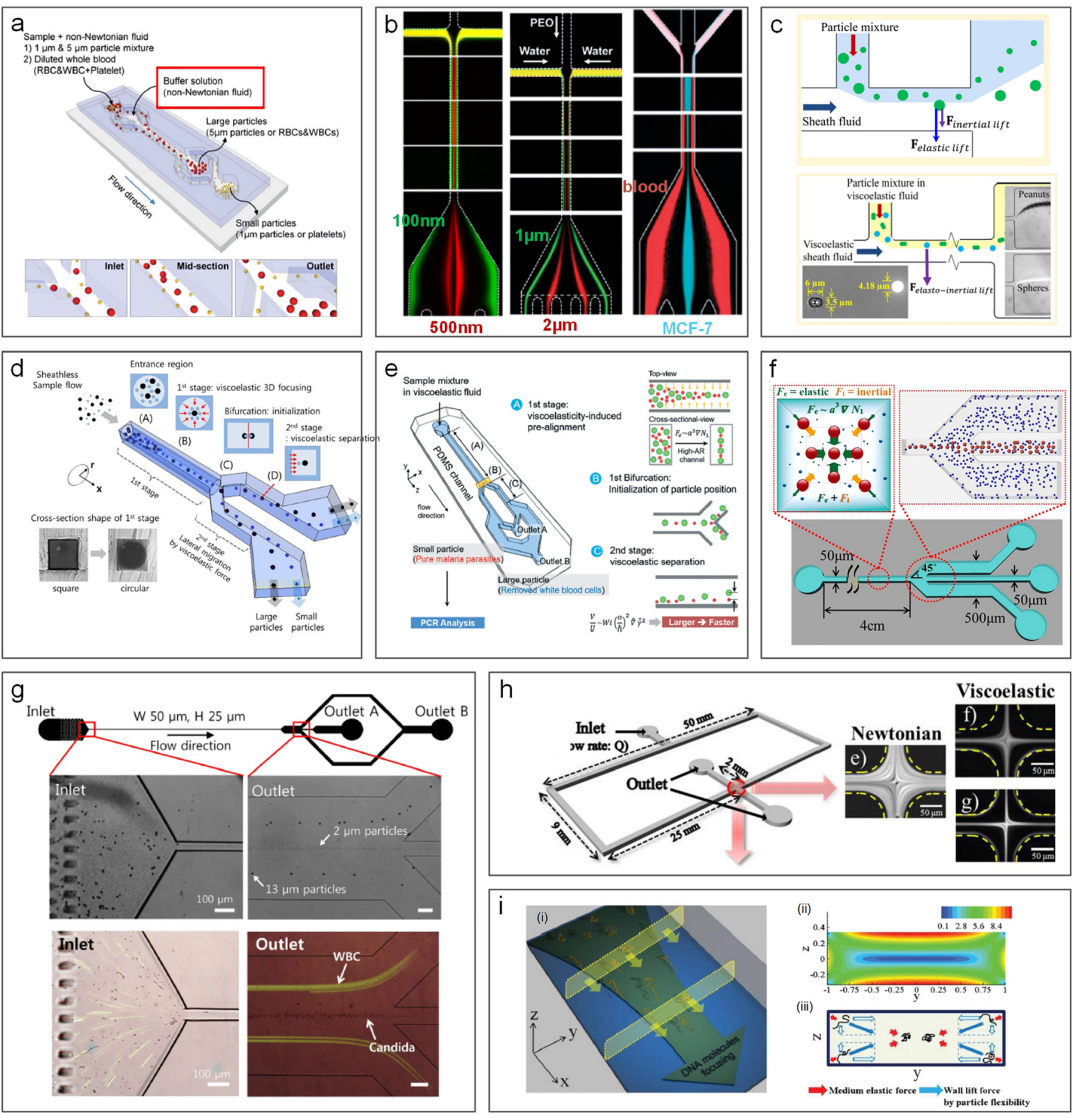
Viscoelastic microfluidics for particle sorting and other applications. (a) Separation of 1 *μ*m and 5 *μ*m diameter particles (or platelets and blood cells) in a square coflow microchannel using viscoelastic flow. Reproduced with permission from Nam *et al.*, Lab on a Chip **12**(7), 1347–1354 (2012). Copyright 2012 Royal Society of Chemistry.^216^ (b) Three coflow channels used for separation of 500 and 100 nm diameter particles,^206^ 1 *μ*m and 2 *μ*m diameter particles,^217^ and MCF-7 and blood cells.^246^ Reproduced with permission from Liu *et al.* ACS Nano **11**(7), 6968–6976 (2017). Copyright 2017 American Chemical Society.^206^ Reproduced with permission from Tian *et al*., Lab on a Chip **17**(18), 3078–3085 (2017). Copyright 2017 Royal Society of Chemistry.^217^ Reproduced with permission from Tian *et al.*, Lab on a Chip **18**(22) 3436–3445 (2018). Copyright 2018 Royal Society of Chemistry.^246^ (c) Combination of viscoelastic flow and pinched flow fractionation (PFF) for size-based^136^ and shape-based^137^ particle separation. Reproduced with permission from Lu and Xuan, Anal. Chem. **87**(12), 6389–6396 Copyright 2015 American Chemical Society.^136^ Reproduced with permission from Anal. Chem. **87**(22), 11523–11530 (2015). Copyright 2015 American Chemical Society.^137^ (d) Sheathless separation of particles in viscoelastic flow using a microchannel with round and square cross sections. Reproduced with permission from Nam *et al.*, J. Chromatogr. A **1406**, 244–250 (2015). Copyright 2015 Elsevier.^215^ (e) Separation of malaria parasites from WBCs in a two-segment channel with a high aspect-ratio cross section.^247^ Reproduced with permission from Nam *et al.*, Lab on a Chip **16**(11), 2086–2092 (2016). Copyright 2016 Royal Society of Chemistry.^247^ (f) Particle filtration in a square microchannel using elastic and inertial forces.^209^ Reproduced with permission from Ahn *et al.*, Chem. Eng. Sci. **126**(14), 237–243 (2015). Copyright 2014 Elsevier. (g) Sheathless separation of particles and cells in viscoelastic flow.^248^ Reproduced from Nam *et al.*, Sci. Rep. **9**(1), 3067 (2019). Copyright 2019 Authors licensed under a CC BY 4.0.^248^ (h) Measurements of monitoring cell deformability using viscoelastic single-stream focusing.^249^ Reproduced with permission from Cha *et al.*, Anal. Chem. **84**(23), 10471–10477 (2012). Copyright 2012 American Chemical Society.^249^ (i) DNA focusing in a rectangular channel based on elastic force and flexibility-induced force.^250^ Reproduced with permission from Kim *et al.*, Lab on a Chip **12**(16), 2807–2814 (2012). Copyright 2012 Royal Society of Chemistry.^250^

Elastic lift force dominates particle migration in such flows when fluid inertia is negligible (*Re* ≪1 and *El* >1).^205,214^ In this case, particles migrate laterally into low shear rate regions, which determine focusing positions of particles flowing in a microchannel. As a result, particles tend to focus into a single position (3D-focusing) located in the channel central axis of a circular microchannel where the shear rate is the lowest [Fig. 19(a)],^214^ despite the fact that the focusing quality could vary depending on different elastic molecules used. In a square microchannel, due to the asymmetric distribution of shear, regions near the four corners also exhibit a stable equilibrium position in addition to the channel central axis [Fig. 19(b)].^205^ Thus, five focusing positions can be observed in such channels as shown by Yang *et al.*^205^ and Seo *et al.*^214^ In a rectangular microchannel, as first demonstrated by Leshansky *et al.* in 2007,^211^ particles migrate toward the central plane of a low aspect ratio channel, where a broad, particle-dense band forms. Particle separation was demonstrated using elastic force in a device consisting of both circular and square channel segments [Fig. 20(d)], but throughput was low (<0.05 *μ*l/min).^215^

Introducing inertial force into a viscoelastic flow system offers twofold benefits in terms of particle focusing and separation. On one hand, non-negligible inertia means a higher flow rate and thus higher processing throughput. On the other hand, the interaction of inertial and elastic forces determines the focusing pattern of particles and therefore provides distinct particle focusing behaviors that can be useful for separation (elastoinertial focusing and separation).^205^ One of the most pronounced differences between inertialess and inertial viscoelastic flows on particle migration is in square channels where single-file 3D focusing can be achieved with the assistance of inertial forces [Fig. 19(c)].^203,205^ Due to the wall induced lift force, particles near the four corners of a square channel are pushed toward the channel center, leading to the elimination of the four focusing positions and entrainment of all particles in the channel axis (3D-focusing).

Since both inertial and elastic forces are highly size-dependent, elastoinertial flow systems have been successfully used for passive particle and cell separations (Fig. 19). Ahn *et al.*^209^ took advantage of the centripetal migration behavior of particles in a square microchannel for separating 2.3 and 4.5 *μ*m particles. PEO solution was used to induce viscoelasticity of the medium, and their flow rate was up to 80 *μ*l/min where inertial force acted simultaneously with elastic force to drive particles toward their equilibrium positions in the channel central axis. As smaller particles move slower due to weaker driving forces, larger 4.5 *μ*m particles reached the channel center ahead of 2.3 *μ*m particles, leading to good separation with 96% recovery for larger particles.

The synergetic interaction of elastic and inertial forces can also lead to distinct focusing positions of different particles in a rectangular microchannel, which can be readily employed for sheathless particle and cell separation. Liu *et al.* showed focusing of 15 *μ*m particles into two streams flanking the central focused stream of 5 *μ*m particles in a rectangular microchannel with an aspect ratio of 2.^204^ Such a size-based focusing pattern was then utilized for separation of MCF-7 cells from red blood cells with an efficiency of 91%. The same scheme with a smaller channel (10 *μ*m height) was also used for separation of *E. Coli* from RBCs in PEO solution with a throughput of ∼2 *μ*l/min. Nam *et al.*^216^ demonstrated a coflow microfluidic device [Fig. 20(a)] achieving a recovery rate >99% for both 1 *μ*m and 5 *μ*m particles at an optimal flow rate of 4.5 *μ*l/min. In this device, PEO flow was injected into the channel and fractured the sample flow into two streams near sidewalls. In this flow configuration, the faster migration of larger particles crossed the flow interface into the clean buffer stream, leading to the clean separation of the two particles. Separation with a recovery rate of 99% was also demonstrated in separation of platelets from highly diluted blood.^216^ Later, the same coflow configuration was used by Tian *et al.*^217^ to achieve a high-resolution separation of 1 *μ*m and 2 *μ*m particles and MCF-7 cells.^246^ Such elastoinertial effects have also been coupled with pinched flow fractionation (PFF) for enhanced particle separation lately^137,210^ [Fig. 20(c)]. Additional demonstrations include the use of viscoelastic flow for particle filtration in square microchannels^209^ as well as sheathless separation of particles^248^ and measurements of cell deformability.^249^ In addition to microparticles and cells, smaller biomolecules can be separated and focused as well. For example, Nam *et al.*^247^ reported separation of malaria parasites from WBCs in a two-segment channel, while Kim *et al.*^250^ reported DNA focusing in a rectangular channel.

While most viscoelastic work has been done in straight channels, curved channels introduce Dean force and thus provide an additional force for the particle migration dynamics. In 2013, Lee *et al.*^218^ first showed focusing and separation of particles suspended in PEO solution flowing in a spiral microchannel. They observed focusing near the outer wall for 10 *μ*m particles and close to the centerline for 1.5 *μ*m particles. Dean force and elastic force were considered responsible for the displacement of focusing positions outward as compared to inertial focusing.^89^ The exact physics underlying such a phenomenon remains unclear despite a recent effort proposing a six-stage focusing model.^219^ A complete separation of these two particles was readily demonstrated in this device by adding four outlets. Similar to straight channels, spiral channels with viscoelastic flows are found to be preferable for manipulating small particles with sizes down to ∼100 nm as demonstrated in a double-spiral channel where a mixture of 100 nm and 2 μm particles was separated with an efficiency >95%.^207^

In summary, focusing and separation in viscoelastic flows is an important addition to the existing inventory of particle separation methods. Viscoelastic manipulation in microchannels is of great interest considering the universally non-Newtonian property of bodily fluids that are critical in healthcare management. The ability of 3D focusing, which is generally difficult to achieve in other passive microfluidic systems, is especially useful in cytometry applications. More importantly, size-based focusing due to elastic force remains effective for particles with sizes down to a few hundreds of nanometers,^206,207^ suggesting promising applications in separation of macromolecules such as DNA [Fig. 20(i)] and extra-small bioparticulates such as exosome. It is advantageous over inertial separation, which is preferred for microscale particle manipulation, and over PFF^46^ whose throughput is roughly a magnitude of order smaller than viscoelastic systems, despite the general requirement of elasticity enhancement, which contaminates sensitive samples.

## IX. SORTING BY SHEAR INDUCED DIFFUSION

While most of the developed microfluidic systems have been designed for separation from diluted samples, separation of particles from highly concentrated suspensions (e.g., whole blood) is preferred in real-world applications. For example, isolation of target cells directly from whole blood^57^ is favored as throughput can be tremendously enhanced and sample preparation is minimal. Due to the complex physics such as strong particle-particle interaction, most of the current microfluidic approaches (e.g., inertial devices) are not applicable for separation in the concentrated sample. Filtration based on porous membranes, including classical membrane filtration^220^ and crossflow filtration,^159^ is one of the few microsystems that can handle the concentrated sample like whole blood despite their poor recovery rate (<50%) and low throughput. As a result, separation of particles/cells from a highly concentrated biosample remains challenging in the field of microfluidic separation science.

The phenomenon of shear induced diffusion (SID) sheds light on addressing the aforesaid challenge. Migration due to SID was first observed by Gadala‐Maria and Acrivos.^221^ It arises from the collision of particles in concentrated suspensions in sheared flows,^222,223^ and it is different from Brownian diffusion.^224^ The strong particle-particle interaction, which is adverse and avoided in inertial microfluidics,^44,45^ is the very driving source of net deterministic migration of particles observed in sheared flow of concentrated suspensions.^222,225^ The net migration of particles due to SID is down the concentration gradient and the shear gradient.^222^ Thus, in a microchannel, the migration is directed away from the wall toward the channel center. Such an effect contributes to the pronounced phenomenon of margination in blood microvasculature where red blood cells (RBCs) migrate toward the vascular center leaving the cell-free layer near the vascular wall.^226^ Similarly, SID also leads to resuspension of particles in crossflow filtration systems^224,227^ and defocusing of cells in some microfluidic flow systems.^228,229^

However, SID holds the promise of label-free separation of particles and/or cells from whole blood and other high-concentration samples. Theoretical works^222,230–233^ have suggested the particle size segregation in highly concentrated suspensions as the down-gradient migration of particles scales with the square of particle size (∼*a*^2^). Tirumkudulu *et al.*^234^ observed particle segregation of monodisperse suspension in a sheared flow. Particle segregation in the binary mixture was successfully demonstrated experimentally in macro- and microchannels,^235,236^ where larger particles were found to be enriched in the channel center. Although very few experimental works have been reported in the literature, separation of platelets from RBCs was possible using the SID effect in microvasculature-sized channels.^237–239^

**FIG. 21.**
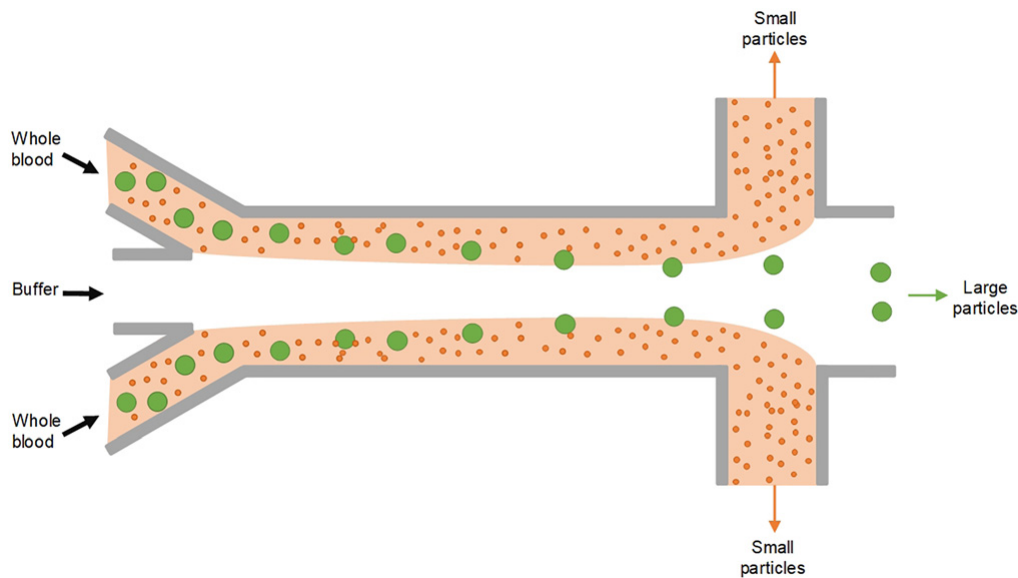
Sorting by shear-induced diffusion (SID). Flow of highly concentrated samples (e.g., whole blood) is split by a faster moving buffer flow in the middle inside a microchannel, giving rise to the effect of shear-induced diffusion for faster toward-center migration of larger particles inside the concentrated side flows.^57^

**FIG. 22.**
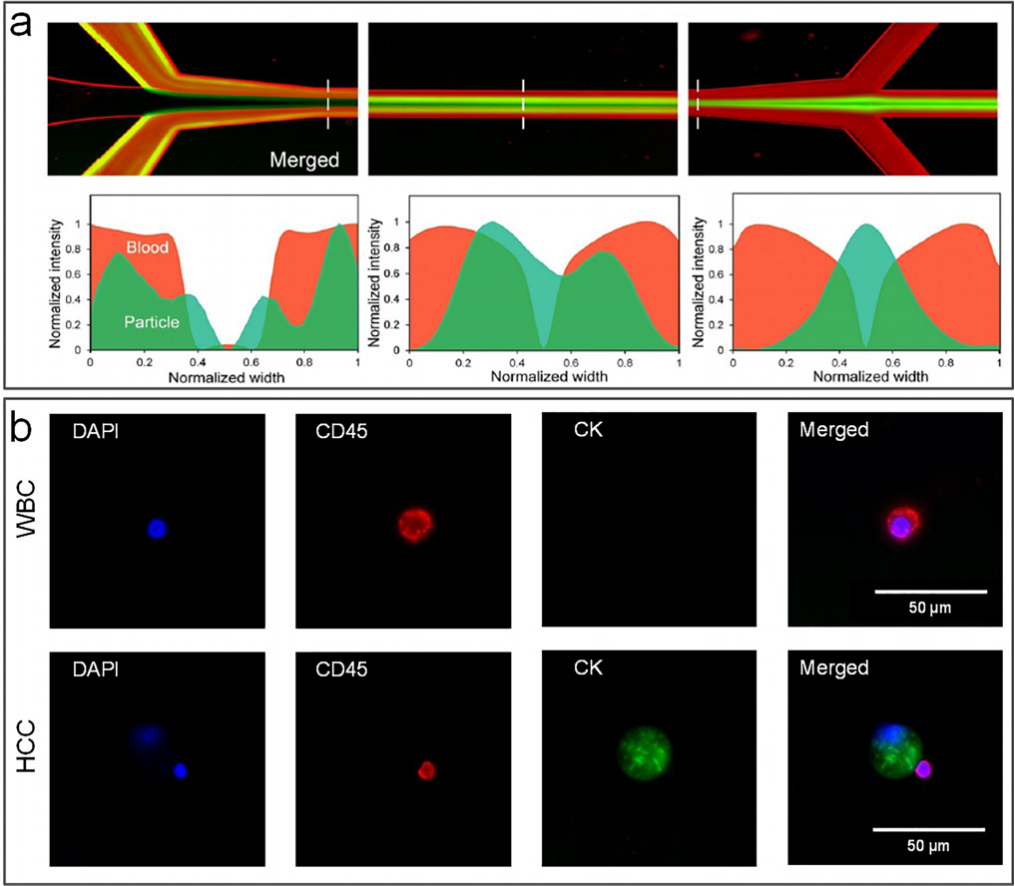
Separation by shear-induced diffusion (SID). (a) 18.7 *μ*m diameter particles (green) migrating away from the blood side-stream (red) and being focused in the middle of the microchannel.^57^ (b) Circulating hepatocarcinoma cell (HCC) and white blood cell (WBC) separated from patient blood using the SID effect.^57^ All images are reproduced from Zhou *et al.*, Sci. Rep. **8**(1), 9411(2018). Copyright 2018 Authors licensed under a CC BY 4.0.^57^

Recently, our group is the first to successfully demonstrate isolation of particles and circulating tumor cells directly from untreated whole blood using the SID effect (Figs. 21 and 22).^57,58^ We engineered the flow configuration inside a microfluidic channel where a Newtonian buffer flow was flanked by two whole blood streams. Such a multiflow configuration generates a concentration gradient across the flow interfaces between blood and buffer. The concentration gradient was coherent with the shear gradient, so that the effect of SID was tremendously enhanced. Particles and cells were found to rapidly migrate away from blood stream toward the buffer. Due to the strong size-dependence of SID, larger particles and cells (e.g., CTCs) migrated faster than smaller RBCs, and thus, cell separation was achieved in a 1-cm channel [Fig. 22(b)]. The performance of our label-free separation is superior with an efficiency of ∼90% at extreme high throughput (10^6^–10^7^ cells/s).^57^ The results are very promising in clinical applications such as rare cell separation despite the fact that much work is required to suppress RBC diffusion. Considering the size variations of RBCs and WBCs, preferential migration of WBCs is possible under the SID effect as recently demonstrated in our other work^240^ where enrichment of larger WBCs was achieved directly in the flow of unprocessed whole blood. Although the size and throughput (volumetric flow rate) ranges for this technique are eclipsed by inertial microfluidics (Fig. 1), the ability to work with unprocessed highly concentrated samples rather than diluted samples is a significant advantage.

## X. CONCLUSIONS AND PERSPECTIVES

With significant attention being paid to the development of microfluidic systems for sorting of microparticles, it is important to understand progress made and the persisting challenges. The passive, label-free methods of particle separation are versatile in mechanism and flexible in particle size range and offer a wide range of throughputs. These critical aspects make passive sorting systems competitive with their active counterparts; yet, they are generally simpler in structure, easier to fabricate, and lower in cost and do not demand skillful operators. This is attributed to the nature of separation mechanisms, which include interactions of particles with flow (e.g., inertial microfluidics), with channel walls or structures (e.g., DLD), with fluid (e.g., viscoelastic flow), and with other particles (e.g., SID) or their combinations (e.g., PFF, HDF, and CFF). Passive label-free devices are capable of processing macro-, micro-, and nanoparticles, with throughputs from nanoliter per minute to milliliter per minute or higher (Fig. 1). Often, a flow injection device is the only one needed for the operation of the passive microfluidic chips, making them particularly favorable in resource-limited regions or circumstances. Simple structures of these devices also enable them to be readily integrated with either upstream sample pretreatment or downstream analysis microfluidic components. All these intriguing traits make the passive label-free devices one of the indispensable building-blocks of lab-on-a-chip systems.

A wide range of methods for passive label-free sorting has been developed for particles with dissimilar physical properties such as size, density, shape, and deformability. The size difference is the most common physical marker used in these methods. Although different methods have their own target range of particle size, together they cover almost the entire spectrum of common particle sizes, from nanometers to millimeters, meeting the need of a wide range of applications. For example, inertial focusing was first discovered in separation of millimeter-sized silica particles,^65,66^ but has since been adapted and widely explored for sorting microparticles, cells, and bacteria. SID, CFF, and HF also work well in this size range. In the past few years, DLD, PFF, and viscoelastic flow-based separation methods have shown their capability of separation of particles down to 20 nm.^179^ Bioparticles such as exosomes have now, too, been successfully separated from other particulate components using these passive methods.^206^

Performance of these passive devices is very promising in terms of throughput, efficiency/recovery, and purity. The throughput spans from nanoliter per minute to microliter per minute or higher depending on the separation mechanism, generally increasing as the target particle size becomes larger (Fig. 1). A smaller channel is necessitated to differentiate smaller particles. For examples, devices based on the principles of PFF, HDF, and CFF are designed for sorting of microparticles of a few microns and typically operate with flow rates in the nanoliter per minute to microliter per minute range, while the inertial and SID devices are capable of operating at more than 100-fold higher flow rates for separating particles above 10 *μ*m in diameter. Most of these label-free devices are able to offer high separation efficiency/recovery (>90%) with some tradeoffs such as lower throughput, requirement of buffer flow, and addition of elasticity enhancer. Among them, high-purity (>90%) separations have also been achieved in inertial, DLD, and viscoelastic devices.

While passive particle separation methods offer excellent performance, they are not without limitations. When compared to their active counterparts, such as acoustic separation,^241^ these methods are less flexible in terms of on-demand activation of separation and tunability of the separation parameters, such as the cut-off size. Since passive devices are free of external force and controls, separation generally begins when proper flow conditions are reached, and thus, on-demand control is not available in these approaches. On the other hand, variation of the separation cut-off size is, in fact, possible by tuning flow conditions in some of the passive methods, such as PFF and HDF, where the change in the flow rate ratio of the sample and buffer flows may provide some degree of flexibility in adjusting the cut-off size.^46,47^ The cut-off size in other techniques is usually dictated by the channel designs, with new devices needed if a different cut-off size is desired. In this regard, a combination of passive and active methods to achieve separation in a complex circumstance is one of the future directions in meeting the real-world needs. For example, a hybrid device consisting of DLD fractionation, inertial focusing, and magnetic-activated sorting components was developed for successful separation of CTCs from the patient blood sample.^242^ A better fundamental understanding of these systems will be necessary to enable integration into more sophisticated and useful platforms.

While new devices are continuously being developed for particle separation, much effort has been made to adapt particle separation platforms for manipulation and separation of bioparticles, including cells,^13^ bacteria,^15^ exosomes,^206^ and even macromolecules such as DNA.^210^ Polymer microparticles can be wonderful surrogates for bioparticles since they are easily available commercially, free from requirements of cell culture, and typically homogeneous in physical properties. As a result, they are widely used for developing and characterizing new sorting devices. However, they are not bioparticles that are flexible and highly heterogeneous. Translating particle separation platforms into cell manipulation devices can require a tremendous amount of effort in taking consideration of properties of biological samples. Developing new particles that better mimic bioparticles can be very helpful. On the other hand, in addition to the size and density, particle shape, deformability, and even surface roughness might be further exploited for developing new label-free passive devices to tackle challenges of novel applications in biomedicine, industry, and beyond.
